# Cross-section without factors: a string model for expected returns

**DOI:** 10.1080/14697688.2024.2357189

**Published:** 2024-06-11

**Authors:** Walter Distaso, Antonio Mele, Grigory Vilkov

**Affiliations:** † Imperial College, South Kensington Campus, London SW7 2AZ, United Kingdom; ‡ USI Lugano, Swiss Finance Institute and CEPR, Via Buffi 13, 6900 Lugano, Switzerland; § Frankfurt School of Finance & Management, Adickesallee 32-34, Frankfurt, Germany

**Keywords:** String models, Correlation premium, Premium for correlation risk, Cross-section of returns, Big stocks, Arbitrage pricing, Implied correlation

## Abstract

Many asset pricing models assume that expected returns are driven by common factors. We formulate a model where returns are driven by a string, and no-arbitrage restricts each expected return to capture the asset's granular exposure to all other asset returns: a correlation premium. The model predicts fresh properties for big stocks, which display higher connectivity in bad times, but also work as correlation hedges: they contribute to a negative fraction of the correlation premium, and portfolios that are more exposed to them command a lower premium. The string model performs at least as well as many existing linear factor models.

## 1. Introduction

The inability of the CAPM to explain the cross-section of expected returns has led to a proliferation of models driven by factors that have recently been the focus of criticism and renewed rigorous statistical scrutiny (see, e.g. Harvey *et al.* 2016). This paper proposes a new arbitrage pricing model in which the cross-section of expected returns links to arguably one amongst the simplest concepts in financial economics: correlation. The distinguishing feature of our approach is that we avoid making reference to factors while explaining asset correlations. Instead, correlations of each asset return with all remaining asset returns are the building block in our framework. That is, in our model, correlations do not result from the assumption of exogenously given ‘pricing factors’. Rather, all correlations are the primitives of the model, and they jointly determine the whole set of no-arbitrage restrictions amongst all asset returns.

Correlation has a long history in asset pricing, although the typical approach has predominantly been to model asset returns in frameworks where correlation and volatility are intimately related. Consider, for example, the seminal Merton (1971) model, in which asset returns are driven by Brownian motions. In that model, the assets correlations are pre-determined by the assumptions made on the assets betas; that is, the price of correlation risk is a function of the ‘lambdas’. Ideally, instead, we would like to disentangle the price of correlation from these lambdas, that is, we would like to disentangle volatility from correlation.

An alternative model is one in which asset returns are driven by shocks that enable one to separate volatility from correlation. We rely on random field models, or stochastic string models, to think about correlation as being determined in this independent way.^1^ Random field models were introduced in finance by Kennedy (1994, 1997) to model the term structure of interest rates, and Goldstein (2000) and Santa-Clara and Sornette (2001) provide extensions or a more general framework in this domain. Tsoulouvi (2005) applies random field models to derivative pricing. Our paper analyzes how random field models can be employed to explain the cross-section of the expected equity returns. Compared to other approaches, ours proposes, then, a new way to model asset returns. Our model is not built up around factors (be they observed or not). We model assets correlations directly, as explained. String models are particularly useful to achieve this goal.

The model works as follows. Asset returns are driven by the realizations of a string. These realizations lead asset returns to co-move, and these co-movements become sources of priced risk: for any asset, the co-movements of its returns with all remaining asset returns receive a compensation. We derive the arbitrage restrictions amongst all asset returns and characterize this compensation: the expected excess return on each asset is the sum of the correlations of this return with all the remaining returns, weighted by some ‘premium function’.

Thus, the expected excess return on any asset reflects an average premium required to compensate for the asset returns granular exposure to all remaining returns. We term the result *correlation premium*. We test whether, indeed, the cross-section of the expected returns is explained by the cross-section of the correlation premia. We find that the model provides a reasonable match of the cross-section of the expected returns, for a number of portfolios sorted through book-to-market, momentum and additional standard characteristics, at least comparable to well-known four- or five- linear factor models (e.g. Fama and French 2015). Furthermore, our model displays additional properties regarding returns predictability and the time-series of assets correlations, both realized and risk-adjusted, as we now explain.

In principle, our model does not require time-varying correlations: even if asset correlations were all constant, the cross-section of the expected excess returns would be a set of non-zero correlation premia. However, in practice, correlations change over time. We model time-variation in these correlations as being driven by a pro-cyclical state variable,^2^ such that correlations increase in bad times, i.e. for low realizations of this state variable. The cross-section of correlation premia and, then, the expected excess returns, are predictable, driven by the state variable. We reconstruct the dynamics of the state variable as a by-product of the model estimation method, based on moment conditions solved in closed-form. The model predicts that, for many portfolios, the cross-section of expected excess returns are countercyclical and asymmetrically related to market conditions: they increase more in bad times than they decrease in good times. We also discuss instances where this relation is reversed: in these cases, some assets may be particularly good hedges in bad times, and portfolios that are particularly exposed to them may command premia that decrease in times of increased market correlations. We find that big size stocks display such properties. More generally, we find that big stocks contribute to a negative fraction of the average correlation premium, such that portfolios that are more exposed to them command a lower premium.

Our moment conditions are based on time-series properties including both realized and option-implied correlations. The model, then, provides additional predictions regarding the random nature of assets correlations. In particular, the risk of changing correlations may lead, and our empirical findings suggest that they do lead, to a premium for *correlation risk*, the difference between risk-adjusted (i.e. option-implied) and realized correlations on S&P 500, a ‘global premium for correlation risk’.^3^

Our model predicts that realized correlations and premia for correlation risk are inversely related. In other words, risk-adjusted correlations move, on average, less than realized correlations in reaction to a changing market environment. This conclusion rationalizes the framework of analysis in the empirical literature of option-implied heterogenous correlations (see, e.g. Buss and Vilkov 2012, Mueller *et al.* 2017 in the foreign exchange space) and it, thus, stands, as a fact that differs from the evidence available from equity volatility markets (reviewed in Section 4.4), by which volatility risk premia are countercyclical. We emphasize that we provide a theoretical model for the premium for correlation risk and that this model also provides predictions on the term structure of premia for correlation risk.

Remarkably, then, our model is able to fit both the premium for correlation risk resulting in derivative markets (on S&P 500), and cross-sections of asset returns that are not directly related to S&P 500. For example, the model is given a comfortable support within the international stock universe, such as the global ME-BTM 5×5 portfolio. Therefore, the model displays potential to explain premia for other asset classes, by just relying on our global premium for correlation risk. In one of the technical appendixes, we focus on supplying additional evidence in the equity space, on a variety of S&P 500 sectors and index-based portfolios. The evidence confirms that our model performance is at least as good as many well-known linear factor models.

Our paper links to several strands of the asset pricing literature. First, and perhaps most important, our paper offers a complementary view to the rich field of factor modeling and testing of the cross-section of expected returns. Since Ross' (1976) seminal paper on arbitrage pricing, the number of published factors has exceeded several hundreds (as reported by Harvey *et al.* 2016). As a result, researchers now carefully concentrate on designing tests to evaluate the asset pricing implications of new factors (see, e.g. Feng *et al.* 2020, amongst others). Our contribution is complementary to this new trend, as we are not proposing new factors, but simply developing a granular account of asset correlations.

By investigating the cross-sectional pricing implications of a string model, we also provide a fresh interpretation of size characteristics (Banz 1981) and the related SMB factor (Fama and French 1993).^4^ We find that in bad times, big stocks are those all other stocks become more connected to, as discussed; because big stocks are also safer than others, assets that are more exposed to big stocks command a lower premium. Small stocks display opposite properties. Thus, a size premium may be seen as a ‘correlation wedge’ that small stocks have with respect to big stocks.

We model the riskiness of a security by relating this same security returns to all other available securities returns. This property, ‘connectivity’, suggests a parallel with the empirical literature of networks in finance, whereby firms in the financial marketplace are interlinked through network effects. A number of papers study the effects of firm-level shocks and their propagation through the economic system (see, e.g. Acemoglu *et al.* 2012, Barrot and Sauvagnat 2016, Herskovic 2018; amongst others). Network effects have also been suggested to explain contagion in financial markets (see, e.g. the early survey of Allen and Babus (2009)) or correlated trading (e.g. Colla and Mele 2010, Ozsoylev *et al.* 2014), and motivated new and several gauges of systemic risk (e.g. Billio *et al.* 2012) as well as asset pricing models that incorporate network effects (Billio *et al.* 2017). Our approach differs from these models due to our emphasis on modeling stochastic correlations based on strings rather than on the traditional input-output network models.

Modeling correlation in financial markets has been the focus of an extensive research agenda over the last decades. Engle (2009) provides an early survey on methods and applications. Our model, based on strings, treats the *dynamics* of correlations in a simple, parsimonious way, assuming correlations are driven by a common, unobserved force. While simple, our model deals with correlation dynamics under both the physical and risk-neutral probabilities, and predicts an empirically plausible premium for correlation risk. Note that the literature on the premium for correlation risk in equity and option markets is quite large and goes back to at least Driessen *et al.* (2009). Buss and Vilkov (2012), Buraschi *et al.* (2014) and Mueller *et al.* (2017) provide relatively more recent contributions, and Faria *et al.* (2022) contain a long list of additional relevant contributions to this topic. Finally, our paper assigns correlation a central role in explaining asset exposure and cross-sectional pricing properties, which go beyond those already studied in option markets. An area of future research is to integrate good parametric and non-parametric models of correlations into our string-based asset pricing framework.

The paper is organized as follows. The next section contains high level assumptions and general no-arbitrage restrictions. Section 3 provides model specifications for the purpose of empirical work. Section 4 develops cross-equation restrictions and contains our empirical results. Section 5 concludes. Appendix 1 contains technical details omitted from the main text, Appendix 2 develops model extensions, and Appendix 3 provides additional empirical evidence not discussed in the main text.

## 2. Asset prices as strings

### 2.1. Primitives

We consider a market with a continuum of assets in (0,1), and assume that each asset return is exposed to all remaining asset returns through the realization of a ‘string’. Previous models with a continuum of assets include those formulated by Al-Najjar (1998) in a static exact factor framework and Gagliardini *et al.* (2016) in a conditional approximate factor setting, amongst others. Our approach is novel precisely because we are not relying on any factor structure, but on strings.

The basic economic intuition underlying a string model for asset returns is that, in this model, each asset return is driven by its own source of uncertainty without implying that all asset returns are uncorrelated. Precisely, let $P_{t}(i)$ be the price of the *i*-th asset at *t* and $D_{t}(i)$ be its instantaneous dividend. We assume that the realized returns on each asset-*i* are solutions to

(1) $$\begin{array}{rl} d{\tilde{R}}_{t}\left(i\right) & \equiv \frac{dP_{t}\left(i\right)+D_{t}\left(i\right)dt}{P_{t}\left(i\right)}\\ & =E\left(y_{t},i\right)dt+\sigma \left(y_{t},i\right)dZ_{t}\left(i\right),\;i\in \left(0,1\right), \end{array}$$

where ${\tilde{R}}_{t}(i)$ is the cumulative return on asset-*i*, $Z_{t}(i)$, the string, is a process continuous in *i* and *t*, and such that $E(dZ_{t}(i))=0$, $\mathrm{var}(dZ_{t}(i))=dt$, and $\mathrm{cov}(dZ_{t}(i)\;dZ_{t}(j))=\rho (y_{t},i,j)\;dt$, for some function *ρ* taking values in (−1,+1), and some state vector $y_{t}$, to be discussed below; the volatility term, σ(y,i) is a continuous function of y and *i*, and ρ(y,i,j), a ‘string correlation function’, is also continuous; finally, E(y,i) is the expected return, determined below (see Proposition 2.1). The function σ(y,i) summarizes the asset-*i* return exposure to how the very same asset return co-varies with all remaining asset returns. It, thus, plays a role similar to the familiar ‘beta’ in traditional factor models.

The notable feature of the model is that returns are risky because the realization of the string leads all asset returns to co-move; in standard models, instead, asset returns co-move, driven by the realization of common factors. In the next section, we explain how the random fluctuations of the string become priced sources of risk, that is, how the expected return on any asset relates to the price of risk of correlation with all other assets. Furthermore, Section 2.4 explains that our model is quite distinct from the standard CAPM, which also predicts that each asset expected returns relate to all other assets' returns (through the market portfolio).

What makes a string model a good model compared to a standard Brownian model? The property mentioned at the beginning of this section: each asset return is driven ‘by its own shock’ and, yet, each asset return may well be correlated with all other asset returns. This property, we now explain, enables us to free up the notion of correlation from that of volatility. Note, indeed, that according to the string model (1), the (square of) volatility of any asset-*i* is

(2) $${\mathrm{vol}}^{2}(d{\tilde{R}}_{t}\left(i\right))\equiv \sigma^{2}\left(y_{t},i\right)dt,$$

and the correlation between any two assets *i* and *j* is

(3) $$\mathrm{corr}(d{\tilde{R}}_{t}\left(i\right),d{\tilde{R}}_{t}\left(j\right))\equiv \rho \left(y_{t},i,j\right).$$

That is, a string model relies on two distinct definitions of volatility and correlation: volatility and correlation are disentangled. This property does not hold for a Brownian model. To review this, consider an asset market driven by Brownian motions, by which

(4) $$d{\tilde{R}}_{t}\left(i\right)=E_{b}\left(y_{t},i\right)dt+v\left(y_{t},i\right)dW_{t},\;i=1,\ldots ,m,$$

where $W_{t}$ is a *d*-dimensional standard Brownian motion, $E_{b}(y_{t},i)$ is the expected return for asset-*i* and, finally, $v(y_{t},i)$ is the exposure of the asset-*i* returns to $W_{t}$, i.e. volatility. In this model, it is impossible to have each asset return driven ‘by its own Brownian motion’, except of course in the uninteresting case where asset returns are all mutually uncorrelated. The correlation between asset *i* and *j* returns is indeed

$$\mathrm{corr}(d{\tilde{R}}_{t}\left(i\right),\;d{\tilde{R}}_{t}\left(j\right))\equiv \frac{\sum_{\ell =1}^{d}v_{\ell }\left(y_{t},i\right)v_{\ell }\left(y_{t},j\right)}{\left\|v\left(y_{t},i\right)\right\|\left\|v\left(y_{t},j\right)\right\|},$$

where $v_{\ell }(\cdot ,j)$ denotes the ℓ-th element of vector v(⋅,i). Note, further, that the correlation matrix is degenerate as soon as *m*>*d*.

As already pointed out by Santa-Clara and Sornette (2001), it is very difficult to specify both volatilities and correlations in a Brownian model, without restricting any of these quantities: the assumptions on $v_{\ell }(\cdot ,\cdot )$ simultaneously determine both of them. Consider, for example, a market where the volatilities of two assets, say assets *a* and *b*, are $v(y_{t},a)=[v_{1a}\;0_{d-1}]$ and $v(y_{t},b)=[v_{1b}\;v_{2b}\;0_{d-2}]$, for three constants $v_{1a},$ $v_{1b}$ and $v_{2b}$, and where $0_{l}$ is a *l*-dimensional vector of zeros. The correlation between these assets returns and the squared volatility of asset *b* are

$$\begin{array}{rl} & \mathrm{corr}(d{\tilde{R}}_{t}\left(a\right),\;d{\tilde{R}}_{t}\left(b\right))=\frac{v_{1b}}{\sqrt{v_{1b}^{2}+v_{2b}^{2}}}\;\mathrm{and}\\ & {\mathrm{vol}}^{2}(d{\tilde{R}}_{t}\left(b\right))=\left(v_{1b}^{2}+v_{2b}^{2}\right)dt. \end{array}$$

That is, asset returns volatilities determine asset returns correlations. By contrast, a string model always makes volatility and correlation two separate objects: see equations (2) and (3).

To illustrate a more complex case, assume that correlations are random but volatilities are constant, as with the string models that we focus on empirically (see Section 4). Equation (4) is consistent with these assumptions when (i) at least one element of $v(y_{t},i)$ is random *and* (ii) $\left\|v(y_{t},k){\|}^{2}=\sum_{\ell =1}^{d}v_{\ell }^{2}(y_{t},k\right)$ is constant for all *k*. Conditions (i)-(ii) are very difficult to satisfy, but may hold very simply with equation (1), as σ(y,i) and ρ(y,i,j) may potentially be driven by different sets of state variables.

Finally, and returning to the description of our string model, one may formulate several assumptions regarding the state vector y. For example, y is another string in one extension of our model developed in Appendix A.3. In our empirical work, we shall assume it is a diffusion process, solution to

$$dy_{t}=b\left(y_{t}\right)dt+\Sigma \left(y_{t}\right)dW_{t},$$

for some vector and diffusion matrix b and Σ. The role of $y_{t}$ in this paper is to generate time-variation in assets' return correlations–not in asset returns, which are driven by strings. In other words, we are considering a string model for the first moments of asset returns, with (for simplicity) a more standard formulation regarding higher moments. We shall rely on this state vector mostly to model the joint behavior of asset correlations. We now provide a description of a pricing kernel that enables one to derive cross-sectional restrictions on each asset expected return.

### 2.2. The pricing kernel

In the absence of arbitrage, there exists a pricing kernel $\xi_{t}$ that prices all the assets. We assume that it is solution to

(5) $$\frac{d\xi_{t}}{\xi_{t}}=-r\left(y_{t}\right)dt-\int_{0}^{1}\phi \left(y_{t},i\right)dZ_{t}\left(i\right)di-\lambda \left(y_{t}\right)dW_{t},$$

where *r* is the instantaneous interest rate, λ is a vector valued function, including the unit prices of risk related to the fluctuations of the Brownian motion $W_{t}$, and $\phi (y,i{)}_{i\in (0,1)}$ is the collection of the unit prices of risk related to the fluctuations of the string $Z_{t}(i{)}_{i\in (0,1)}$. We assume that these prices of risk are continuous functions of the state vector y and *i*. From now on, we focus on the asset pricing implications of the pure string component and, accordingly, we shall refer the collection $\phi (y,i{)}_{i\in (0,1)}$ as the *string premium*. Appendix 2 contains extensions that allow for the existence of a priced Brownian risk. We now turn to the cross-sectional restrictions on each asset expected return.

### 2.3. Conditional CAPM and the correlation premium

In a frictionless market, the expected return on each asset-*i* satisfies the following standard restriction

(6) $$\begin{array}{rl} E\left(y_{t},i\right)dt & =E\left(\frac{dP_{t}\left(i\right)+D_{t}\left(i\right)dt}{P_{t}\left(i\right)}\right)\\ & =r\left(y_{t}\right)dt-\mathrm{cov}\left(\frac{dP_{t}\left(i\right)}{P_{t}\left(i\right)},\frac{d\xi_{t}}{\xi_{t}}\right),\;i\in \left(0,1\right). \end{array}$$

We have

(7) $$\begin{array}{rl} & \mathrm{cov}\left(\frac{dP_{t}\left(i\right)}{P_{t}\left(i\right)},\;\frac{d\xi_{t}}{\xi_{t}}\right)\\ & =-E\left(\sigma \left(y_{t},i\right)dZ_{t}\left(i\right)\int_{0}^{1}\phi \left(y_{t},j\right)dZ_{t}\left(j\right)dj\right)\\ & =-\sigma \left(y_{t},i\right)\int_{0}^{1}\phi \left(y_{t},j\right)E\left(dZ_{t}\left(i\right)dZ_{t}\left(j\right)\right)dj\\ & =-\sigma \left(y_{t},i\right)\left(\int_{0}^{1}\phi \left(y_{t},j\right)\rho \left(y_{t},i,j\right)dj\right)dt. \end{array}$$

Replacing these results into equation (6) leaves the following restrictions on the cross-section of expected returns:

Proposition 2.1 **(Correlation premium)**

The expected return $E(y_{t},i)$ on asset-*i*, i∈(0,1), satisfies

(8) $$E\left(y_{t},i\right)-r\left(y_{t}\right)=C\left(y_{t},i\right),$$

where

(9) $$C\left(y_{t},i\right)\equiv \sigma \left(y_{t},i\right)\int_{0}^{1}\phi \left(y_{t},j\right)\rho \left(y_{t},i,j\right)dj.$$

The term $C(y_{t},i)$ in this proposition summarizes the evaluation of the asset-*i* granular exposure to the market, and we are referring to it as the *correlation premium* for asset-*i*. The proposition provides a novel theory of the cross-section of the expected returns, based on this correlation premium. Equation (8) tells us that each asset expected excess return *i* is the premium required to compensate an investor for the exposure of the asset-*i* return to all remaining asset returns. The contribution of asset-*j* return to the premium for asset-*i*, when the state is y, is σ(y,i)ϕ(y,j)ρ(y,i,j)dj. That is, ρ(⋅,i,j) is the correlation between asset-*i* and asset-*j* returns, correlation arising from the realization of the string; ϕ(⋅,j) is the unit risk premium that compensates for any risk correlated with the asset-*j* return; finally, σ(⋅,i) defines the size of the overall exposure of the asset-*i* return to the whole string, as explained in Section 2.1.

To illustrate Proposition 2.1, consider the following heuristic example based on a *J*-asset market, as summarized by table 1. Consider, say, asset-*i*. Its returns are exposed to the risk of co-movements with returns on asset-1, a risk summarized by the correlation, $\rho (y_{t},i,1)$; then, $\sigma (y_{t},i)\rho (y_{t},i,1)$ is the risk of co-variation that returns on asset-*i* have with returns on asset-1. We term this co-variation ‘exposure’, in analogy with standard asset pricing terminology. Now, there are obviously *J* such exposures resulting from the realization of the string, including the variation of the very same asset-*i* returns. According to the model, each of these exposures receives a compensation. The correlation premium is the average premium, C, as summarized by table 1, i.e. the counterpart to equation (9) in this heuristic example.

**Table 1.** This table provides a heuristic construction of the expected return required to hold any asset *i*. The second column indicates how asset-*i* is exposed to fluctuations of any asset *j*. The second column is the unit risk premium required to bear a given exposure to any asset *j*, $\phi_{j}\equiv \phi (y_{t},j)$. The correlation premium, C, is the average of the exposures weighted by the unit risk premia.

	Exposure to asset-*j*	Compensation	Premium
*j* = 1	$\sigma (y_{t},i)\rho (y_{t},i,1)$	$\phi_{1}$	$\sigma (y_{t},i)\rho (y_{t},i,1)\phi_{1}$
···	···	···	···
*j* = *J*	$\sigma (y_{t},i)\rho (y_{t},i,J)$	$\phi_{J}$	$\sigma (y_{t},i)\rho (y_{t},i,J)\phi_{J}$
			$C=\sigma (y_{t},i)\frac{1}{J}\sum_{j=1}^{J}\rho (y_{t},i,j)\phi_{j}$

This example illustrates that, in the model, exposures are the counterparts to the familiar ‘betas’ in standard factor models. That is, betas are asset returns sensitivities to changes in common factors; instead, in our model, exposures result from the asset returns sensitivities to changes in all the asset returns that arise through the realization of the string. Similarly, compensations are the counterparts to ‘lambdas’. But while lambdas are unit risk premia relating to the fluctuations of common and exogenous factors, compensations are, in our model, unit premia rewarding an investor for how each asset return co-varies with all remaining asset returns: there exists, then, a compensation for the exposure to each asset return in the assets universe. In Section 3, we formulate assumptions that help deal with these infinite dimensional problems, rendering our model tractable for empirical purposes. Prior to this formulation, we highlight some properties of the model that help distinguish it from the standard CAPM.

### 2.4. Relations with the standard CAPM

In the standard CAPM, the expected excess returns on each asset link to the market portfolio and, hence, to *all* assets' expected returns. What makes our model different? *Heterogeneity*: the property that the realized returns on each asset (to which any asset returns may be exposed to) are risks that require ‘their own’ compensation. In our model, then, the premium for investing in any risky asset compensates for the granular exposure of this asset returns to shocks in all other asset returns, as equations (8)-(9) predict. By contrast, the standard CAPM predicts that the cross-section of risk-premia reflects exposures to a *common* factor (the market portfolio), such that each of the assets in the market portfolio requires the same compensation for risk, mechanically adjusted for its market cap.

To highlight these differences, we analyze the cross-sectional difference of the expected returns by focusing on two arbitrary assets. For simplicity, we consider the simple case in which correlations, volatilities and unit string-premia are constant. Note that the standard CAPM predicts that, for any asset-*i*,

$$\Pi \left(i\right)\equiv E\left(i\right)-r=\frac{\mathrm{cov}\left(i,M\right)}{\sigma_{M}^{2}}\left(E_{M}-r\right),$$

where cov(i,j) denotes the covariance between two portfolio returns, *i* and *j*, ω(i) is market cap of asset-*i* and, finally, $E_{M}\equiv \int_{0}^{1}\omega (i)E(i)\;di$, and $\sigma_{M}^{2}\equiv \iint_{i,j\in [0,1{]}^{2}}\omega (i)\sigma (i)\rho (i,j)\omega (j)\sigma (j)di\;dj$. In order to focus on our theme in this paper–correlations–assume, next, that volatilities are constant in the cross-section, σ(i)=σ (say). Then, the difference in premia for any two assets, a,b∈(0,1), is

$$\Pi \left(a\right)-\Pi \left(b\right)=\sigma \int_{0}^{1}\omega \left(j\right)\cdot \lambda \left(\mathrm{corr}\left(a,j\right)-\mathrm{corr}\left(b,j\right)\right)dj,$$

where $\lambda =(E_{M}-r)/\sigma_{M}$ is the standard unit risk premium, the Sharpe ratio. That is, the difference in premia is a weighted sum of the difference in correlations of the two assets' returns with respect to all remaining assets' returns. But crucially, in this model, each of the assets' returns receives the same compensation, *λ*.

Our string model works differently. By equations (8)–(9), the differences in premia required to invest in *a* and *b* are

$$C\left(a\right)-C\left(b\right)=\sigma \int_{0}^{1}\phi \left(j\right)\left(\mathrm{corr}\left(a,j\right)-\mathrm{corr}\left(b,j\right)\right)dj.$$

For the string, the difference in correlations with asset-*j* is weighted by ϕ(j), which varies over j∈(0,1): the returns of each security contribute to the overall risk of any asset and requires ‘its own’ compensation, which is proportional to ϕ(j)corr(⋅,j), as explained in table 1. In other words, in the string model, there is a risk-premium related to each security *j*; in the standard CAPM, there is a unique premium, *λ*, for all securities.

Now, analytically, and under all the assumption in this section, the string model collapses to the CAPM under the razor's edge condition by which ϕ(j)=λ⋅ω(j), for all *i*. However, this condition bears little economic meaning: the terms ϕ(j) are string-premia (that is, compensations for risk), whereas, ω(i) are, more mechanically, cap weights. The next section explains that, in Appendix 2, we have developed a consumption-based string model where the string-premium relating to any asset-*j* may be increasing in the dividend share of this asset. While dividend shares could be a possible proxy for firm size, they are however quite distinct from market caps. Even more crucially, the condition that high string-premia increase with firm size is strongly rejected by data (see Section 4) as our empirical analysis suggests a quite complicated structure for these string-premia. In Section 3, we proceed with the formulation of additional modeling details of the string-premia in the general random environment, $\phi (y_{t},i)$, which we rely on while estimating our cross-sectional restrictions.

### 2.5. Consumption-based correlation risk

What would a consumption-based model predict regarding the correlation premium? In Appendix 2, we study Euler's conditions in a string economy and identify the correlation premium that results in this economy. This premium is proportional to a weighted average of all the assets' dividend volatilities (weighted by the asset dividends' shares), where the proportionality factor is the CRRA (see equation (A15)). One additional property of this economy is that the returns on each asset depend on the asset specific shock (as in equation (1)) but also, indirectly, on the realization of the whole string (see equation (A16)). This property arises due to market clearing, as equilibrium asset prices do in general depend on the realization of the whole share process, a complication well-known since previous work on consumption-based multi-asset models (see, e.g. Menzly *et al.* 2004). This property justifies an extension of the model in this section, whereby any asset-*i* returns are driven by a ‘compound string’, that is, by the realization of a convex functional of the whole string (i.e. not only by $dZ_{t}(i)$). The reader will find details regarding formulation, solution and economic interpretation of the compound string model in Appendix 2. We now formulate additional assumptions regarding the main model we focus in this paper–the Conditional CAPM string model of this section–with the purpose of taking it to data.

## 3. A model with random correlations

This section provides model specifications that account for the salient empirical properties of (i) asset return correlations and (ii) the premia required to bear time-variation in these correlations.

**Figure 1.**
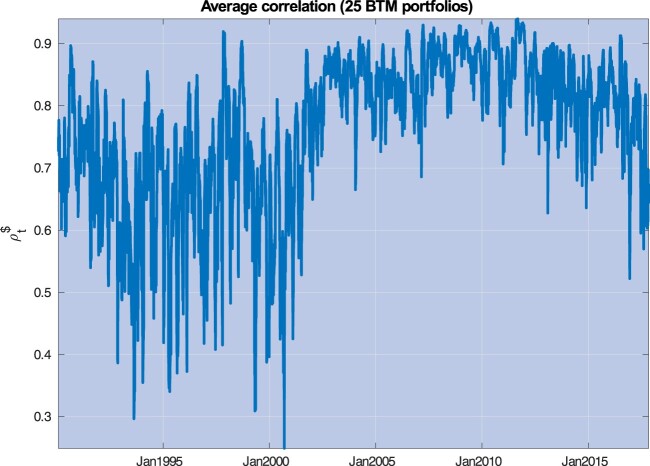
This picture depicts the average correlation exposure for 25 Size and Book-to-Market sorted portfolios, defined as $\rho_{t}^{\$}=\frac{1}{n}\sum_{i=1}^{n}\sum_{j=1}^{n}\rho_{t}^{\$}(i,j)$, where $\rho_{t}^{\$}(i,j)$ is the realized correlation between portfolios *j* and *i*, obtained through a rolling window equal to 22 days.

It is well-known that asset correlations do indeed vary over time (see, e.g. Figure 1). Initially, however, it is instructive to focus on our model implications in the simple case where correlations, variances and premia are all constant. Assume, then, that for all i,j∈(0,1),

$$\begin{array}{rl} & \sigma \left(y_{t},i\right)=\sigma_{i},\;\rho \left(y_{t},i,j\right)=\rho \left(i,j\right),\;\phi \left(y_{t},j\right)=\phi_{o},\\ & \lambda \left(y_{t}\right)=\lambda_{o}, \end{array}$$

for some constants $\sigma_{i}$, ρ(i,j), $\phi_{o}$ and a vector of constants $\lambda_{o}$. Given these assumptions, Proposition 2.1 predicts that the expected excess returns on each asset-*i* are

(10) $$\begin{array}{rl} & E\left(y_{t},i\right)-r\left(y_{t}\right)=C\left(i\right),\\ & C\left(i\right)=\phi_{o}\sigma_{i}\underset{\equiv \rho_{i}\left(\mathrm{global}\;\mathrm{correlation}\;\mathrm{exposure}\right)}{\underbrace{\left(\int_{0}^{1}\rho \left(i,j\right)dj\right)}.} \end{array}$$

We call $\rho_{i}$ *global correlation exposure (GCE)* for asset-*i*, consistent with terminology in Section 2.3 (see table 1): the risk premium on asset-*i* equals the product of a risk exposure, $\sigma_{i}\rho_{i}$, times the unit price of string-risk, $\phi_{o}$. We refer to $\rho_{i}$ as ‘global’ because it is the average correlation of asset-*i* returns with all other asset returns. This decomposition of the expected returns is neat, but obtains due to the assumption that the unit prices of risk are constant in the cross-section. We now generalize the insights from this basic model and account for both time-variation in correlations and cross-sectional variations in the unit risk premia.

**Figure 2.**
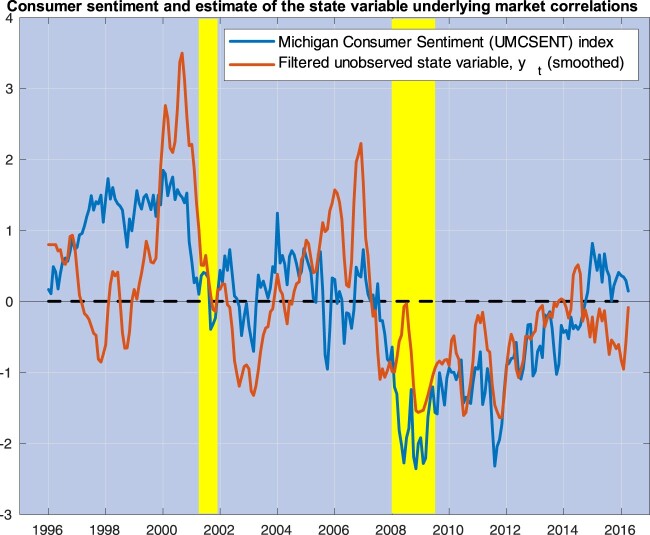
This figure plots three-month moving averages of the estimates of $y_{t}$, the state variable driving market correlations (red line), along with the University of Michigan Consumer Sentiment (UMCSENT) index (blue line). Both variables are de-meaned and standardized by their own standard deviations. The yellow shaded areas cover recession episodes as determined by the National Bureau of Economic Research.

### 3.1. A factor model of asset correlations

Figure 1 summarizes well-known evidence regarding time-variation in asset correlations. We consider 25 Size and Book-to-Market sorted portfolios and calculate realized correlations for each portfolio pair through one-month rolling windows estimates. Consider the empirical counterpart to the global correlation exposures $\rho_{i}$ in equation (10), $\rho_{t}^{\$}(i)=\frac{1}{n}\sum_{j=1}^{n}\rho_{t}^{\$}(i,j)$, where $\rho_{t}^{\$}(i,j)$ denotes the realized correlation between portfolios *j* and *i*, and *n* = 25. We find that nearly 90% of the variability in these correlation exposures is explained by the first principal component. Figure 1 plots the average correlation exposure, defined as $\rho_{t}^{\$}=\frac{1}{n}\sum_{i=1}^{n}\rho_{t}^{\$}(i)$. Section 4.2 provides a detailed description of the historical episodes leading to the main spikes experienced by additional measures of correlation exposures (see figure 5 and table 3).

The fact that a large portion of the correlation exposures is driven by a single principal component suggests that a parsimonious model may help explain time-variation in these correlations. We now proceed with such a model while still assuming that correlation is priced in accordance with the string model of Section 2. We assume that the asset correlations are driven by a diffusion process $y_{t}\equiv y_{t}$, a scalar. To keep the model as simple as possible, we still assume that the exposures to strings are constant and independent of *i*, i.e. $\sigma (y_{t},i)=\sigma_{i}$; and we assume that the string correlation function is

(11) $$\rho \left(y_{t};i,j\right)=\varrho_{0}\left(i,j\right)+\varrho_{1}\left(i,j\right)e^{-y_{t}},$$

where $\varrho_{0}(i,j)$ and $\varrho_{1}(i,j)$ are matrix coefficients independent of time, and such that $\varrho_{0}(i,i)=1$ and $\varrho_{1}(i,i)=0$, and $y_{t}$ is solution to a square root process

(12) $$dy_{t}=\kappa \left(m-y_{t}\right)dt+\eta \sqrt{y_{t}}\;dW_{t},$$

for three positive constants *κ*, *m* and *η*. Under standard parameter restrictions, $y_{t}$ stays strictly positive, hence, this specification for $y_{t}$ bounds $\rho (y_{t},i,j)$ to be inside the unit circle as soon as $|\varrho_{0}(i,j)+\varrho_{1}(i,j)|<1$.

Note that a string model for pairwise correlations may well be an alternative to the modeling assumptions underlying equations (11) and (12). However, we adopt the assumptions of this section because our main focus is on the cross-section of expected returns and last, but not least, for the sake of simplicity. The formulation in (11) is both analytically convenient and intuitive: correlations are made up of a constant and a dynamic component, with sensitivities to changes in $y_{t}$, $\varrho_{1}(i,j)$, which vary across all asset pairs. For the purpose of identifying the model, we need to fix the sign of $\varrho_{1}(i,j)$, and we work with $\varrho_{1}(i,j)\geq 0$. We shall, then, refer $y_{t}$ to as a pro-cyclical variable: correlations are down when $y_{t}$ is up. Note, however, that correlations are not always linked to the business cycle. There might be correlation spikes during periods of financial distress, but such episodes may well be transitory and occurring during a favorable phase of the business cycle, as in the instances identified and discussed in Section 4 (see Figure 5).

In other words, our model merely defines bad times as times of high correlations. Is there a more precise interpretation of our unobserved factor? Figure 2 plots monthly estimates of $y_{t}$ obtained in Section 4 against the University of Michigan Consumer Sentiment (UMCSENT) index; both series are de-meaned and standardized by their own standard deviation. This index is known to track reasonably well the long swings in consumers' ‘sentiment’ around business cycle fluctuations. Our extracted variable correlates with this index at approximately 60%. In previous work, Corradi *et al.* (2013) have noticed that long-run movements in stock market fluctuations link to this index. It is remarkable that market correlations (Wall Street) and consumer sentiment (Main Street) are also statistically quite closely related.

### 3.2. The correlation premium

The next corollary summarizes cross-section restrictions resulting from the assumptions formulated in Section 3.1.

Corollary 3.1 **(One-factor correlation premia)**

Assume that the correlation function satisfies equation *(11)*, where $y_{t}$ is solution to equation *(12)*, and that each asset return variance is constant and equal to $\sigma_{i}^{2}$ for asset-*i*. Then, the expected excess returns in Proposition *2.1* (equations *(8)*-*(9)*) are

(13) $$\begin{array}{rl} & E\left(y_{t},i\right)-r\left(y_{t}\right)=C\left(y_{t},i\right),\\ & C\left(y_{t},i\right)=\sigma_{i}\int_{0}^{1}\phi \left(y_{t},j\right)\left(\varrho_{0}\left(i,j\right)+\varrho_{1}\left(i,j\right)e^{-y_{t}}\right)dj. \end{array}$$

Thus, the cross-section of the expected excess returns is driven by a single, pro-cyclical state variable, $y_{t}$. Moreover, under conditions on $\phi (y_{t},j)$ discussed in a moment, expected excess returns are decreasing and convex in $y_{t}$, that is, they are countercyclical and react asymmetrically to $y_{t}$: they increase in bad times more than they lower in good. This property is known to hold, empirically, at the aggregate level, and at a business cycle frequency (see Mele 2007). However, a similar property may not necessarily hold for the model in this paper because correlations may well spike in good times, as discussed in the previous section.

Furthermore, some assets may display a few desirable properties due to their ability of being more resilient to systemic shocks: while a very few assets may occasionally withstand to a widespread turmoil where there is ‘no place to hide’ (see, e.g. Buraschi *et al.* (2014)), some assets' performance, a subset J say, may still suffer relatively much less than others' in bad times. This property makes these assets natural ‘hedges’: now, asset returns that have more exposure to those in J may require a lower premium. In Section 4, we provide evidence that big stocks display such hedging property, and that some portfolio returns particularly exposed to them may, then, be even pro-cyclical, under conditions (see Section 4.3).

We now proceed with specifying three functional forms for the string premia that we use in our empirical work.

(I) *Constant premia.* The correlation premium is constant both in time and in the cross-section, that is, $\phi (y_{t},j)\equiv \bar{\phi }$. In this case, the correlation premium in equation (13) collapses to

(14) $$C\left(y_{t},i\right)=\bar{\phi }\sigma_{i}\underset{\equiv \rho_{i}\left(y_{t}\right)\;\left(\mathrm{dynamic}\;\mathrm{GCE}\right)}{\underbrace{\left(\varrho_{0}\left(i\right)+\varrho_{1}\left(i\right)e^{-y_{t}}\right)}},$$

where $\varrho_{q}(i)=\int_{0}^{1}\varrho_{q}(i,j)\mathrm{dj}$, *q* = 0, 1. This model specification is a very minimal generalization of the constant correlation model in equation (10), whereby the global correlation exposure (GCE), $\rho_{i}$, is replaced with its dynamic counterpart, $\rho_{i}(y_{t})$. Still, the properties of $\rho_{i}(y_{t})$ play an important role in the interpretation of our empirical results (see Section 4.3).

(II) *Cross-sectional variation.* The correlation premium for shocks on the asset return-*j* links to the dynamic GCE in (14) for the same asset, $\rho_{j}(y_{t})$, according to $\phi (y_{t},j)=\phi_{0}\varrho_{0}(j)+\phi_{1}\varrho_{1}(j)$, for two constants $\phi_{0}$ and $\phi_{1}$. The correlation premium for asset-*i* in Corollary 3.1 is

(15) $$\begin{array}{rl} & C\left(y_{t},i\right)\\ & =\sigma_{i}\int_{0}^{1}\left(\phi_{0}\varrho_{0}\left(j\right)+\phi_{1}\varrho_{1}\left(j\right)\right)\left(\varrho_{0}\left(i,j\right)+\varrho_{1}\left(i,j\right)e^{-y_{t}}\right)dj. \end{array}$$

(III) *Time series and cross-sectional variation.* The correlation premium for shocks on asset-*j* links to $\rho_{j}(y_{t},i)$, according to $\phi (y_{t},j)=\phi_{v0}\varrho_{0}(j)+(\phi_{v1}+\phi_{v2}e^{-y_{t}})\varrho_{1}(j)$, for three constants $\phi_{v0}$, $\phi_{v1}$ and $\phi_{v2}$, such that the correlation premium for asset-*i* is

(16) $$\begin{array}{rl} C\left(y_{t},i\right) & =\sigma_{i}\int_{0}^{1}\left(\phi_{v0}\varrho_{0}\left(j\right)+\left(\phi_{v1}+\phi_{v2}e^{-y_{t}}\right)\varrho_{1}\left(j\right)\right)\\ & \;\left(\varrho_{0}\left(i,j\right)+\varrho_{1}\left(i,j\right)e^{-y_{t}}\right)dj. \end{array}$$

The rationale behind the specifications in (II) and (III) is the following. A parsimonious modeling assumption is that the premium $\phi_{j}(y_{t},j)$ for exposure to asset returns-*j* reflects information on the dynamic GCE for the very same asset, $\rho_{j}(y_{t})$. In these formulations, then, this premium reflects both the unconditional part of $\rho_{j}(y_{t})$, i.e. $\varrho_{0}(j)$, and the exposure of $\rho_{j}(y_{t})$ to movements in the state variable $y_{t}$, $\varrho_{1}(j)$. The difference between (II) and (III) is that the latter reflects both cross-sectional information (i.e. on $\varrho_{0}(j)$ and $\varrho_{1}(j)$) and time series information (i.e. on the state of $y_{t}$). Note that the specification (III) encompasses (II), namely for $\phi_{v2}=0$.

The following proposition gathers the expressions for the cross-section of the unconditional expected returns in the three specifications formulated above.

Proposition 3.2 **(Unconditional correlation premia)**

The unconditional expected returns predicted by (I) the constant premia model, (II) the cross-sectional variation model, and (III) the time series and cross-sectional variation model, are

(17) $$E\left[C\left(y_{t},i\right)\right]=\left\{ \begin{array}{ll} \bar{\phi }\sigma_{i}\left(\varrho_{0}\left(i\right)+\varrho_{1}\left(i\right){\bar{Y}}_{\left(1\right)}\right) & \left(I\right)\\ \sigma_{i}\int_{0}^{1}\left(\phi_{0}\varrho_{0}\left(j\right)+\phi_{1}\varrho_{1}\left(j\right)\right)\\ \left(\varrho_{0}\left(i,j\right)+\varrho_{1}\left(i,j\right){\bar{Y}}_{\left(1\right)}\right)dj & \left(\mathrm{II}\right)\\ \sigma_{i}\int_{0}^{1}[\phi_{v0}b_{0}\left(i,j\right)+\phi_{v1}b_{1}\left(i,j\right)\\ +\phi_{v2}b_{2}\left(i,j\right)]dj & \left(\mathrm{III}\right) \end{array}\right.$$

where $b_{0}(i,j)\equiv A_{0,0}(i,j)+A_{0,1}(i,j){\bar{Y}}_{\left(1\right)}$, $b_{1}(i,j)\equiv A_{1,0}(i,j)+A_{1,1}(i,j){\bar{Y}}_{\left(1\right)}$, $b_{2}(i,j)\equiv A_{1,0}(i,j){\bar{Y}}_{\left(1\right)}+A_{1,1}(i,j){\bar{Y}}_{\left(2\right)}$, $A_{h,q}(i,j)\equiv \varrho_{h}(j)\varrho_{q}(i,j)$, and

$${\bar{Y}}_{\left(\ell \right)}\equiv E\left(e^{-\ell y_{t}}\right)={\left(\frac{2\kappa }{2\kappa +\ell \eta^{2}}\right)}^{\frac{2\mathrm{\kappa m}}{\eta^{2}}},\;\ell =1,2.$$

In Section 4, we estimate our string model while relying on its unconditional version predicted by Proposition 3.2, similarly as with standard methodology used with the Conditional CAPM (e.g. Jagannathan and Wang 1996, Lettau and Ludvigson 2001. We now develop additional cross-equation restrictions that we use while estimating the model. We address the question: is $y_{t}$ a source of priced risk?

### 3.3. The premium for correlation risk

**Figure 3.**
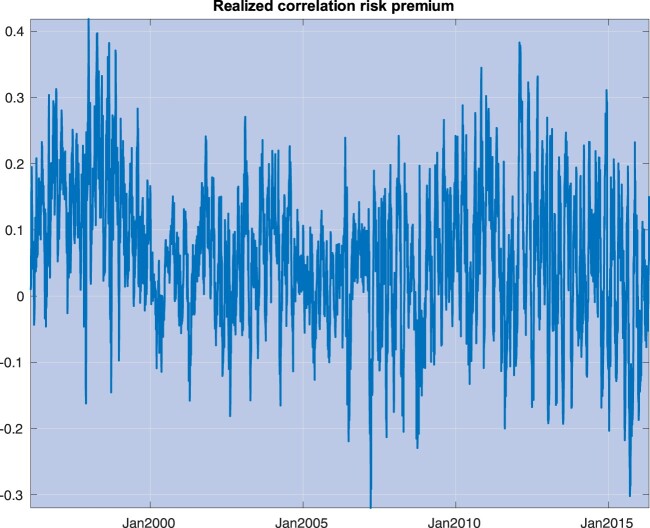
This picture plots the realized premium for correlation risk for S&P 500 stocks, defined as the difference between (i) risk-adjusted expectations of one-month average correlations, and implied by option prices, and (ii) realized correlations, calculated throughout a one-month window.

A key concept that has been extensively investigated in the empirical literature is the premium for *correlation risk*, defined as the difference between the expected integrated correlation under the risk-neutral probability and the physical probability, denoted hereafter as *Q* and *P*, respectively. If correlation was not a priced risk, this difference would always be zero. Figure 3 depicts the *realized* premium for correlation risk for S&P 500 stocks, defined as the difference between option implied integrated correlations (that is, correlations expected under *Q*) and realized correlations (proxies for expectations under *P*). Section 4 contains a detailed description of our input data and computations used in figure 3.

Consistent with the empirical evidence, we assume that time-variation in correlations is a priced risk. Our point of departure is the string correlation function $\rho (y_{t};i,j)$ in equation (11). Let us integrate this function twice with respect to all asset pairs, obtaining the average correlation amongst all asset returns,

(18) $$\rho (y_{t};\varrho )=\iint_{i,j\in {\left[0,1\right]}^{2}}\rho \left(y_{t};i,j\right)di\;dj=\varrho_{0}+\varrho_{1}e^{-y_{t}},$$

where we have defined $\varrho =[\varrho_{0},\varrho_{1}]$ and $\varrho_{q}=\int_{0}^{1}\varrho_{q}(i)\;di$, *q* = 0, 1. The model-implied premium for correlation risk is defined as the difference between the average expected integrated correlation $\rho (y_{t};\varrho )$ in (18) under *Q* and that under *P*

(19) $$P_{t}\equiv \frac{1}{T-t}\left[\int_{t}^{T}E_{t}^{Q}\left(\rho \left(y_{\tau };\varrho \right)\right)d\tau -E_{t}\left(\rho \left(y_{\tau };\varrho \right)\right)d\tau \right],$$

where $E_{t}^{Q}(\cdot )$ denotes the expectation under *Q* given information at time-*t*, and *T*−*t* is a given time horizon.

In words, the premium for *correlation risk* compensates an investor for the fluctuations in the asset correlations. Note, also, that this definition is distinct from the *correlation premium*, i.e. C(⋅,i) in Proposition 2.1, as emphasized in the Introduction (see footnote 3). The correlation premium, C(⋅,i), compensates for any asset return exposure to all remaining asset returns. The premium for correlation risk, $P_{t}$, compensates for randomness in this exposure. Furthermore, note that $y_{t}$, the factor driving this random exposure, is not priced in the cross-section of the expected returns. Appendix B indicates how to proceed under the assumption that $y_{t}$ is also priced in the cross-section of the expected returns. However, to keep the model as simple as possible, we do not consider this extension.

To render equation (19) operational, we specify the unit risk premium for $y_{t}$. We assume that $\lambda (y)=\nu \sqrt{y}$ for some constant *ν*, such that, under the risk neutral probability, *Q*,

(20) $$dy_{t}=\tilde{\kappa }\left(\tilde{m}-y_{t}\right)dt+\eta \sqrt{y_{t}}\;d{\tilde{W}}_{t},$$

where ${\tilde{W}}_{t}$ is a standard Brownian motion under *Q*, and

$$\tilde{\kappa }=\kappa +\nu \eta ,\;\tilde{m}=\frac{\mathrm{\kappa m}}{\kappa +\nu \eta }.$$

Because $y_{t}$ is interpreted as a pro-cyclical variable, we expect, and find, empirically (see Section 4.2), that ν>0, meaning that $y_{t}$ is more frequently in bad times under *Q* than under *P* (see Proposition A.1 in Appendix 1).

Let $\vartheta =[\theta ,\varrho_{1},\nu ]$, where θ=[κ,m,η] denotes the parameter vector under the physical probability. Accordingly, denote with $P_{t}=P(y_{t};\vartheta )$ the model-based premium for correlation risk in equation (19) for a given set of parameter values ϑ. The next proposition, proved in Appendix 1, provides motivation for this notation as well as some properties of this premium for correlation risk.

Proposition 3.3 **(Premium for *correlation risk*)**

Assume that the premium related to Brownian fluctuations is $\lambda (y)=\nu \sqrt{y}$. Then, the premium for correlation risk is

(21) $$\begin{array}{rl} & P\left(y_{t};\vartheta \right)\\ & =\frac{\varrho_{1}}{T-t}\int_{t}^{T}\left(u\left(y_{t},\tau -t;\theta ,\nu \right)-u\left(y_{t},\tau -t;\theta ,0\right)\right)d\tau , \end{array}$$

where

(22) $$\begin{array}{rl} & u\left(y,x;\theta ,\nu \right)=a\left(x;\nu \right)e^{-b\left(x;\nu \right)y},\\ & a\left(x;\nu \right)={\left(\frac{2\tilde{\kappa }}{2\tilde{\kappa }+\eta^{2}\left(1-e^{-\tilde{\kappa }x}\right)}\right)}^{\frac{2\mathrm{\kappa m}}{\eta^{2}}},\\ & b\left(x;\nu \right)=\frac{2\tilde{\kappa }e^{-\tilde{\kappa }x}}{2\tilde{\kappa }+\eta^{2}\left(1-e^{-\tilde{\kappa }x}\right)}. \end{array}$$

Moreover, for ν>0, the premium for correlation risk is (i) strictly positive; and is (ii) increasing and concave in $y_{t}$ for all $y_{t}$ lower than some $y_{1}$; and (iii) decreasing and convex in $y_{t}$ for all $y_{t}$ higher than some $y_{2}>y_{1}$.

**Figure 4.**
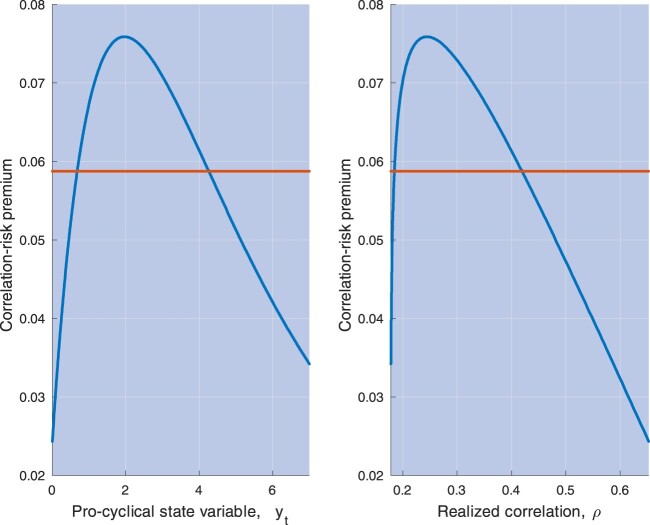
This picture plots the one-month premium for correlation risk $P(y_{t};\vartheta )$ in equation (21) against the state variable $y_{t}$ (left panel) and the average correlation predicted by the model, $\rho (y_{t};\varrho )$ in equation (18) (right panel). Parameter values are set equal to their estimates obtained in Section 4 (see table 2). The red line is the unconditional expected value of the premium for correlation risk predicted by the model, i.e. $E(P(y_{t};\vartheta ))$ in equation (26).

Proposition 3.3 tells us that, provided correlation risk is positively priced, ν>0, the premium for this risk achieves a maximum. In good times, when the pro-cyclical variable $y_{t}$ is high, the premium for correlation risk rises as $y_{t}$ lowers. As times deteriorate further, additional drops in $y_{t}$ lead to a fall in this premium. This fall reflects that fact that, in bad times, correlations under *P* and under *Q* are already very high; because they are obviously both bounded, then, as $y_{t}$ lowers, their difference tends to vanish. These properties are illustrated by the left panel in figure 4, which plots the premium for correlation risk $P(y_{t};\vartheta )$ in equation (21), and its unconditional expectation, based on the parameter estimates obtained in Section 4.

The right panel of figure 4 depicts the premium for correlation risk against the average correlation predicted by the model estimates, $\rho (y_{t};\varrho )$ in equation (18), obtained while varying the state variable $y_{t}$ driving them. The descending part of the curve in this right panel does correspond to the ascending part of the curve in the left panel. The model prediction, then, is that for most values of the instantaneous correlation, correlations and the premium for correlation risk are inversely related, with the premium achieving its maximum when correlation is about as low as 25%.^5^ These predictions are useful because, while $y_{t}$ is not observed, we may estimate correlations and the premium for correlation risk based on observable quantities. Section 4 provides additional details on the testable implications of the model in this dimension, and evidence of a strong negative relation between correlations and the premium for correlation risk, consistent with the model predictions (see figure 12 in Section 4.4).

## 4. Empirical analysis

### 4.1. Data and preparation of variables

#### 4.1.1. Sources

For the model calibration, we require data on a wide panel of individual stocks belonging to a large index with traded options, and also data on a smaller panel of realized returns for a set of test assets. The first large panel is used to estimate the correlation state variable, and the smaller panels are then used to test our cross-sectional predictions. We rely on a daily data sample that runs from January 1996 until April 2016. For the smaller panels, we use returns on standard Fama-French portfolios. We calculate second moments (volatilities, correlations, and factor betas) based on daily returns, and then proceed to estimate risk premia relying on monthly portfolios returns.

As a broad sample of individual stocks, we select all constituents of a market-wide index, namely, S&P500. The composition of S&P500 index is obtained from Compustat and merged with CRSP through the CCM Linking Table using GVKEY and IID to link to PERMNO, following the second best method from Dobelman *et al.* (2014). The data on daily returns and market capitalization are obtained from CRSP, and as a proxy for index weights on each day, we use the relative market cap of each stock in an index from the previous day.

For the cross-sectional tests, we use a number of standard portfolios, sorted by characteristics such as market equity (ME), book-to-market (BTM), investment (INV), operating profitability (OP), momentum (MOM), and reversal (REV). We obtain daily and monthly returns for these portfolios from Kenneth French data library. The cross-sectional pricing results are based on six sets of portfolios, each with 25 assets stemming from different two-way sorting procedures. We use the following data sets: 5×5 ME-BTM, 5×5 ME-INV, 5×5 ME-MOM, 5×5 ME-REV, 5×5 ME-OP, and 5×5 ME-BTM Global portfolios.

We would like our model to deliver not only cross-sectional pricing performance, but also to be consistent with the premium for correlation risk. To help achieve the second task, we rely on option data on the S&P500 index and all its constituents and compute the time series of the implied correlations and the premia for correlation risk, defined below. Implied correlations are estimated by comparing the index variance with the variance of the portfolio of index components. To compute the option-based variables, we rely on the Surface File from OptionMetrics, selecting for each underlying the options with 30, 91 and 365 days to maturity and deltas in the out-the-money range (that is, absolute delta weakly less than 0.5). While the surface data is not suitable for testing trading rules due to extensive inter- and extrapolations of market data, it proves to be a valuable source of information that can be used in asset pricing tests or in generating signals for trading.

#### 4.1.2. Model inputs

The estimation of our model requires calibrating the string correlation function in (11) to its empirical counterparts. We calibrate the model in a way that the correlation state variable $y_{t}$ reproduces model dynamics for the average correlation in (18) and its risk-neutral equivalent (defined in a moment) that match as closely as possible their empirical counterparts. As for these empirical counterparts, we rely on average correlations obtained through the equicorrelation amongst all S&P500 components. Equicorrelation is a useful measure of the average level of market-wide correlations and, hence, it may reasonably be based upon for the purpose of proxying the dynamics of our state-variable. Equicorrelations are computed assuming that, in each day, all pairwise correlations are equal.^6^

Consider a basket of assets with a variance equal to $\sigma_{\mathrm{It}}^{2}$ at time-*t*:

$$\sigma_{\mathrm{It}}^{2}=\sum_{i,j=1}w_{i}w_{j}\sigma_{\mathrm{it}}\sigma_{\mathrm{jt}}\rho_{\mathrm{ij},t},$$

where $w_{i}$ are the asset portfolio weights. Given a time-series of variances of this basket, $\sigma_{\mathrm{It}}^{2}$, of its components $\sigma_{\mathrm{it}}^{2}$, and the index weights, $w_{i}$, equicorrelations are obtained as the single number $\rho_{\mathrm{ij},t}=\rho_{t}$ calculated in each day *t* as

(23) $$\rho_{t}=\frac{\sigma_{\mathrm{It}}^{2}-\sum_{i=1}w_{i}^{2}\sigma_{\mathrm{it}}^{2}}{\sum_{i=1}\sum_{j\neq i}w_{i}w_{j}\sigma_{\mathrm{it}}\sigma_{\mathrm{jt}}}.$$

Note that the resulting correlation matrix of the assets in the basket is positive-definite, provided the equicorrelation is non-negative, which is the case in our empirical implementation of (23). In the sequel, we refer to ‘implied correlation’ for the risk-neutral, and ‘realized correlation’ for the realized equicorrelations.

Option-implied variances are computed as model-free implied variances (Dumas 1995, Britten-Jones and Neuberger 2000, Bakshi *et al.* 2003). We compute realized variances using daily returns and a window length equal to one month. Thus, after plugging the implied or realized variances into equation (23), we end up with the monthly implied or realized correlations, respectively. The premium for correlation risk is calculated as in Driessen *et al.* (2005) as an implied correlation at the end of day-*t* minus 22-day moving averages of the realized correlations under *P* calculated through (23). We denote the estimate of this premium at time-*t* with $P_{t}^{\$}$. Likewise, let $\rho_{t}^{\$}$ denote the realized correlation at time-*t*. As primary data series for calibrating the parameters governing the dynamics of $y_{t}$, we use one-month realized correlation, $\rho_{t}^{\$}$, and such is, then, the time horizon of the corresponding premium for correlation risk, $P_{t}^{\$}$. To calibrate the string correlation function (i.e. $\varrho_{0}(i,j)$ and $\varrho_{1}(i,j)$ in (11)), we calculate equation (23) using realized standard deviations on each single asset. Pairwise correlations are computed from daily returns by relying on standard formulas. Finally, the cross-sectional tests of our models are based on monthly realized excess returns of test portfolios. The excess returns are computed as realized monthly returns minus the one-month Treasury bill rate (from Ibbotson Associates) obtained from the Kenneth French data library.

### 4.2. Cross-equation restrictions and state variable estimates

We develop moment conditions that we use to estimate θ, the parameter vector related to the dynamics of the pro-cyclical state variable $y_{t}$ under *P* (see Section 3), the correlation exposures $\varrho_{0}(i,j)$ and $\varrho_{1}(i,j)$, and the coefficient *ν* of the premium for correlation risk. Finally, we explain how we proceed to recover estimates of the pro-cyclical state variable for each date in our sample.

#### 4.2.1. Matching correlations and the premium for correlation risk

The next proposition provides moment conditions that we use to estimate $\left(\theta ,\varrho_{1}\right)$.

Proposition 4.1 **(Correlation moment conditions)**

For any integer *n*, the *n*-th uncentered unconditional moment of $\rho (y_{t};\varrho )$ is

(24) E(ρn(yt;ϱ))=∑i=0n(ni)ϱ0iϱ1n−i(2κ2κ+(n−i)η2)2κmη2.

For any fixed Δ, the unconditional covariance of $\rho (y_{t};\varrho )$ with $\rho (y_{t+\Delta };\varrho )$ is

(25) $$\begin{array}{rl} & \mathrm{cov}\left(\rho (y_{t};\varrho ),\rho (y_{t+\Delta };\varrho )\right)\\ & =\varrho_{1}^{2}\left[{\left(\frac{4\kappa^{2}}{{\left(2\kappa +\eta^{2}\right)}^{2}-\eta^{4}e^{-\kappa \Delta }}\right)}^{\frac{2\mathrm{\kappa m}}{\eta^{2}}}-{\left(\frac{4\kappa^{2}}{{\left(2\kappa +\eta^{2}\right)}^{2}}\right)}^{\frac{2\mathrm{\kappa m}}{\eta^{2}}}\right]. \end{array}$$

Provided the state variable $y_{t}$ is mean-reverting ( κ>0), the auto-covariance of the integrated correlation, $\rho (y_{t};\varrho )$, is strictly positive and vanishes to zero, eventually. The higher *κ*, the higher the vanishing rate, just as for the original state variable $y_{t}$. Note, also, that *m*, the unconditional mean of $y_{t}$, can be identified with enough moment conditions. Intuitively, the variance of a square root process is level-dependent, such that the whole autocovariance function of $y_{t}$ is level-dependent too.

Proposition 4.1 helps reconstructing the dynamics of $y_{t}$ under the physical probability. Moreover, we may rely on the model-implied premium for correlation risk in Proposition 3.3 and derive additional parameter restrictions. In Appendix 1, we show that the unconditional mean of $P(y_{t};\vartheta )$ is

(26) $$E\left(P\left(y_{t};\vartheta \right)\right)=\frac{\varrho_{1}}{T-t}\int_{0}^{T-t}\left(\bar{u}\left(x;\theta ,\nu \right)-\bar{u}\left(x;\theta ,0\right)\right)dx,$$

where

$$\bar{u}\left(x;\theta ,\nu \right)={\left(\frac{2\tilde{\kappa }\kappa }{2\tilde{\kappa }\kappa +\left(\kappa +\nu \eta e^{-\tilde{\kappa }x}\right)\eta^{2}}\right)}^{\frac{2\mathrm{\kappa m}}{\eta^{2}}}.$$

We use a moment condition based on equation (26) as an additional cross-equation restriction for $\left[\theta ,\varrho_{1}\right]$. Note that we do *not* need this restriction in order to estimate the cross-section of expected returns. However, it helps pinning down the level of the premium for correlation risk to its historical average, through the parameter *ν*. (The horizontal, red lines in figure 4 are the values of $E(P(y_{t};\vartheta ))$ implied by our parameter estimates.) Precisely, let $\zeta =[\theta ,\nu ,\varrho_{0},\varrho_{1}]$ and let *N* denote the sample size. Define

$$h_{N}(\zeta )\equiv \left[\begin{array}{c} E_{N}(\rho_{t}^{\$})-E\left(\rho (y_{t};\varrho )\right)\\ \mathrm{va}r_{N}(\rho_{t}^{\$})-\mathrm{var}\left(\rho (y_{t};\varrho )\right)\\ E_{N}(\rho_{t}^{\$3})-E\left(\rho^{3}(y_{t};\varrho )\right)\\ \left\{\mathrm{co}v_{N}(\rho_{t}^{\$},\rho_{t+\Delta }^{\$}\right)\\ \;-\mathrm{cov}\left(\rho (y_{t};\varrho ),\rho (y_{t+\Delta };\varrho )\right){\}}_{\Delta \in L}\\ E_{N}(P_{t}^{\$})-E\left(P\left(y_{t};\vartheta \right)\right) \end{array}\right],$$

where *N* subscripts indicate empirical moment estimates and, finally, L denotes the set of lags chosen while calibrating the model-implied autocovariance function to its data counterparts: two weeks, one month and two months. Our GMM estimator is obtained as

(27) $${\hat{\zeta }}_{N}=\arg\min_{\zeta }h_{N}(\zeta {)}^{⊺}W_{N}h_{N}(\zeta ),$$

where $W_{N}$ is a weighting matrix that minimizes the asymptotic variance of the estimator, which we estimate, recursively, as ${\hat{W}}_{N}^{-1}\equiv h_{N}({\hat{\zeta }}_{N}{)}^{⊺}h_{N}({\hat{\zeta }}_{N})$.

Therefore, we rely on 7 moment conditions to estimate 6 parameters. Table 2 contains parameter estimates and associated t-statistics. All parameter estimates are highly statistically significant.

**Table 2.** GMM estimates of ${\hat{\zeta }}_{N}$ in (27) and t-stats.

	Estimate	t-stat
$\varrho_{0}$	0.1779	7.23
$\ln\varrho_{1}$	−0.7460	−3.71
*κ*	3.8753	3.81
*m*	2.2423	2.71
*η*	4.1688	2.87
*ν*	3.3518	15.97

#### 4.2.2. Estimates of correlation exposures

To implement cross-sectional estimates of the model in (13), we need to estimate the asset return correlation exposures in equation (11), $\varrho_{0}(i,j)$ and $\varrho_{1}(i,j)$ and, thus, build up estimates of the state. We rely on estimates of $y_{t}$ obtained while minimizing a distance of the model predictions to the data proxies $\rho_{t}^{\$}$ and $P_{t}^{\$}$,

(28) $${\hat{y}}_{t}=\arg\min_{y_{t}}\left(\frac{(\rho_{t}^{\$}-\rho (y_{t};{\hat{\varrho }}_{N}){)}^{2}}{\mathrm{var}(\rho_{t}^{\$})}+\frac{(P_{t}^{\$}-\bar{P}({\hat{\vartheta }}_{N}){)}^{2}}{\mathrm{var}(P_{t}^{\$})}\right),$$

where $\rho (y_{t};\varrho )$ is defined in (18) and $\bar{P}({\hat{\vartheta }}_{N})$ denotes the model counterpart to $P_{t}^{\$}$.^7^ Therefore, we are using option data for the purpose of extracting information on the state variable that drives the assets' correlations. This objective is not strictly needed while only focussing on the cross-section of expected returns–we could have omitted the second term of the minimand in (28). However, option data may provide additional and useful information for the purpose of estimating both correlation premia and the cross-section of expected returns.

Estimates of the correlation exposures, say ${\hat{\varrho }}_{0}(i,j)$ and ${\hat{\varrho }}_{1}(i,j)$ are, then, obtained while regressing data proxies, $\rho_{t}^{\$}(i,j)$ say, onto a constant and $e^{-{\hat{y}}_{t}}$, under the restriction that the coefficient estimates sum up to the GMM estimates in (27), viz

$${\hat{\varrho }}_{q}=\iint_{i,j\in {\left[0,1\right]}^{2}}{\hat{\varrho }}_{q}\left(i,j\right)di\;dj,\;q\in \left\{0,1\right\}.$$

Finally, we use ${\hat{\varrho }}_{0}(i,j)$ and ${\hat{\varrho }}_{1}(i,j)$ in equation (13) and implement cross-sectional estimates of the prices of risk ϕ(⋅) while fitting the unconditional version of the model predicted by Proposition 3.2 in its three versions, as implied by (14), (15), and (16). Section 4.3 discusses results on these estimates.

**Figure 5.**
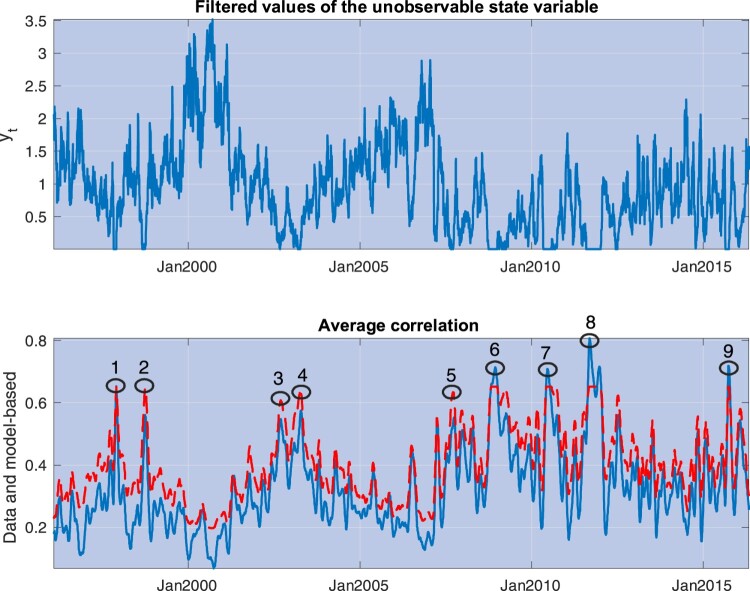
The top panel depicts estimates of the pro-cyclical state variable, $y_{t}$, obtained by matching the model predictions on realized correlations and the premium for correlation risk, as in equation (28). The bottom panel depicts the average correlation in the data (solid, blue line) and the average correlation predicted by the model (dashed, red line). The numbered circles identify events described in table 3.

Figure 5 depicts the estimates of the state obtained through (28) as well as a comparison of the average correlations predicted by the model with those in the data. Model predictions are obtained as 22-day rolling window averages of $\rho ({\hat{y}}_{t};{\hat{\varrho }}_{N})$, and average correlations in the data are obtained in the same way from S&P 500 stocks. The model tracks all the major episodes of spikes in correlations that occurred during our sample period (defined as the nine episodes in which model-based correlations reached their highest levels). Table 3 provides a succinct description of the events leading to these spikes.

**Table 3.** This table provides descriptions of the major events leading to the spikes in correlation depicted in Figure 5.

Event	Date	Description
1	Nov 1997	Asian crisis corrections
2	Sep 1998	Russian financial crisis
3	Sep 2002	Bear market corrections
4	Mar 2003	Iraq War
5	Sep 2007	Subprime crisis
6	Sep 2008	Lehman Brothers bankruptcy
7	Jun 2010	European debt crisis I: Greece bailout
8	Aug 2011	European debt crisis II: spreading
9	Aug 2015	Chinese market corrections

We now turn to provide cross-sectional estimates of the price of risk and discuss the model implications on the cross-section of expected returns.

### 4.3. Cross-sectional pricing

We test the unconditional version of the asset pricing model (13), implied by the three dynamic specifications of the premium for correlation risk predicted by Proposition 3.2: (I) constant premium $\phi (y_{t},j)=\bar{\phi }$ both in time and cross-sections, (II) premium with cross-sectional variation $\phi (y_{t},j)=\phi_{0}\varrho_{0}(j)+\phi_{1}\varrho_{1}(j)$, for two constants $\phi_{0}$ and $\phi_{1}$, and (III) premium with time and cross-sectional variation $\phi (y_{t},j)=\phi_{v0}\varrho_{0}(j)+(\phi_{v1}+\phi_{v2}e^{-y_{t}})\varrho_{1}(j)$, for three constants $\phi_{v0}$, $\phi_{v1}$ and $\phi_{v2}$.

We employ a two-pass Fama-MacBeth (1973) procedure to estimate the coefficients of the string premium ϕ(y,⋅). We estimate these coefficients by regressing the cross-section of the test portfolio realized returns onto the cross-sectionally exogenous variables appearing on the R.H.S. of equation (17) (e.g. $\sigma_{i}\int_{0}^{1}b_{x}(i,j)\;dj$, for *x* = 0, 1, 2, for Model III). Note that the three specifications are all linear in the premium coefficients, which facilitates calculations and statistical inference. Specifically, for each portfolio *i* in a given set of test portfolios, we compute the model-based expected return using as inputs the estimate of the volatility parameter $\sigma_{i}$, the estimates of the unconditional moments of the correlation level ${\bar{Y}}_{\left(\ell \right)}$, ℓ=1,2, and the estimates of the correlation exposures $\varrho_{0}(i,j)$ and $\varrho_{1}(i,j)$. We gauge the overall model fit by comparing the unconditional model-based average returns with the realized returns for the whole sample period. Tables 4 to 6 provide parameter estimates and adjusted- $R^{2}$ for the three models on six sets of test portfolios.

**Table 4.** This table provides parameter estimates of $\bar{\phi }$ in the constant string premium Model I, $\phi (y_{t},j)=\bar{\phi }$ (with t-stats below), and the pricing performance expressed as the average pricing error (*α* is annualized) across a given set of portfolios, and the fit of the model (adjusted- $R^{2}$, ${\bar{R}}^{2}$) from this regression.

	$\bar{\phi }$	*α*	${\bar{R}}^{2}$
5×5 ME-BTM	0.315	0.078	−0.020
	0.540	1.881	–
5×5 ME-INV	0.434	0.073	0.016
	1.242	2.893	–
5×5 ME-MOM	0.136	0.099	−0.035
	0.552	4.711	–
5×5 ME-REV	0.878	0.033	0.196
	3.070	1.442	–
5×5 ME-OP	−0.002	0.101	−0.043
	−0.004	2.978	–
5×5 ME-BTM Global	−0.307	0.103	−0.033
	−0.448	2.267	–

**Table 5.** This table provides parameter estimates of $\phi_{0}$ and $\phi_{1}$ in the cross-sectional variation premium Model II, $\phi (y_{t},j)=\phi_{0}\varrho_{0}(j)+\phi_{1}\varrho_{1}(j)$ (with t-stats below) and the pricing performance expressed as the average pricing error (*α* is annualized) across a given set of portfolios, and the fit of the model (adjusted- $R^{2}$, ${\bar{R}}^{2}$) from this regression.

	$\phi_{0}$	$\phi_{1}$	*α*	${\bar{R}}^{2}$
5×5 ME-BTM	26.377	−15.188	0.181	0.235
	2.646	−2.248	2.618	–
5×5 ME-INV	51.991	−27.727	0.195	0.373
	3.547	−3.345	4.344	–
5×5 ME-MOM	34.776	−17.782	0.145	0.736
	11.921	−11.604	12.728	–
5×5 ME-REV	34.718	−16.770	0.102	0.502
	4.434	−4.156	5.608	–
5×5 ME-OP	86.275	−45.169	0.233	0.795
	10.309	−10.121	11.117	–
5×5 ME-BTM Global	70.577	−38.144	0.219	0.746
	7.191	−7.480	9.251	–

**Table 6.** This table provides parameter estimates of $\phi_{v0}$, $\phi_{v1}$ and $\phi_{v2}$ in the time and cross-sectional variation premium Model III, $\phi (y_{t},j)=\phi_{v0}\varrho_{0}(j)+(\phi_{v1}+\phi_{v2}e^{-y_{t}})\varrho_{1}(j)$ (with t-stats below), and the pricing performance expressed as the average pricing error (*α* is annualized) across a given set of portfolios, and the fit of the model (adjusted- $R^{2}$, ${\bar{R}}^{2}$) from this regression.

	$\phi_{v0}$	$\phi_{v1}$	$\phi_{v2}$	*α*	${\bar{R}}^{2}$
5×5 ME-BTM	−1.492	12.596	−30.290	0.132	0.330
	−0.076	0.696	−1.654	2.042	–
5×5 ME-INV	75.497	−49.833	24.470	0.212	0.417
	3.676	−2.865	1.276	6.003	–
5×5 ME-MOM	37.126	−20.059	2.667	0.145	0.737
	3.543	−2.107	0.255	12.471	–
5×5 ME-REV	27.276	−9.775	−7.894	0.099	0.507
	1.652	−0.673	−0.490	4.836	–
5×5 ME-OP	138.671	−92.983	53.900	0.238	0.891
	11.149	−9.358	5.584	15.532	–
5×5 ME-BTM Global	42.508	−14.140	−25.249	0.218	0.784
	2.669	−1.163	−2.158	9.264	–

For comparison, table 7 provides adjusted- $R^{2}$ for a number of unconditional linear factor models fitted to the same portfolio returns of tables 4 through 6: the CAPM, and 3- (Fama and French 1993), 4- (Carhart 1997) and 5- (Fama and French 2015) factor models. As with our string models, these measures of fit are obtained by fitting average excess returns of the test assets through the average returns predicted by the models. Our Models II and III seem to provide a quite reasonable fit and, with the exception of one case (5×5 ME-INV), certainly better than the linear factor models. Consider the following heuristic calculations. If we average the ${\bar{R}}^{2}$ across all portfolios in table 7, we obtain 0.222 (CAPM), 0.125 (3-F), 0.403 (4-F) and 0.397 (5-F). In comparison, the average ${\bar{R}}^{2}$ in table 6 for the string Model III is 0.611, a performance much better than that of the 4-F model. Appendix 3 contains results regarding S&P 500 sectors and index-based portfolios, and achieves to equally encouraging conclusions: on average, our string models perform essentially the same as the 4-F model, but better than others. We now discuss our results on string models in detail.

**Table 7.** This table provides adjusted- $R^{2}$ from linear factor model regressions across the portfolios analzyed in tables 4 through 6. The four columns provide the adjusted- $R^{2}$ for the CAPM, the 3-F model (market, value, and size), the 4-F model (market, value, size, and momentum), and the 5-F model (market, size, value, profitability, and investment factors).

	CAPM	3-F	4-F	5-F
5×5 ME-BTM	0.168	−0.040	0.186	0.299
5×5 ME-INV	0.130	0.442	0.487	0.536
5×5 ME-MOM	0.348	−0.019	0.697	0.369
5×5 ME-REV	0.216	0.145	0.225	0.195
5×5 ME-OP	0.482	0.105	0.618	0.733
5×5 ME-BTM Global	−0.011	0.117	0.202	0.251

**Figure 6.**
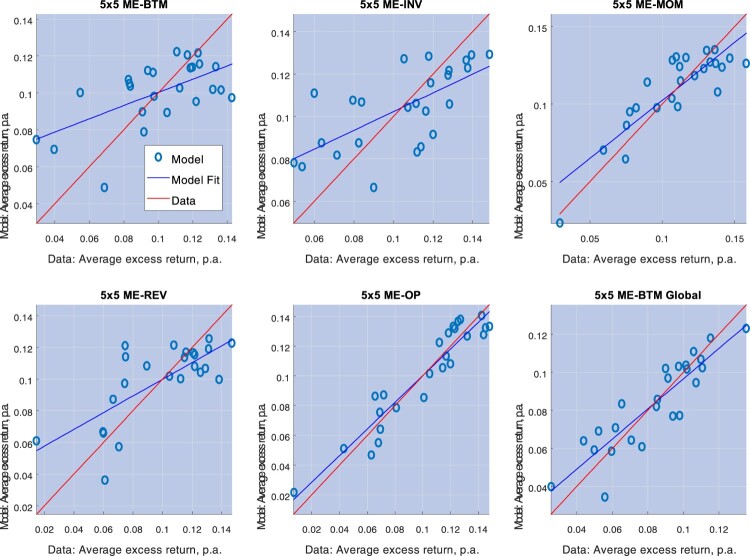
This picture depicts average excess returns and Model III predictions on the unconditional expected excess returns (the unconditional correlation premium of Proposition 3.2), for 5×5 ME-BTM, 5×5 ME-INV, 5×5 ME-MOM, 5×5 ME-REV, 5×5 ME-OP, and 5×5 ME-BTM Global portfolios.

The helicopter view at the models' estimates tells us that the constant risk premium in both cross-sectional and time-series dimensions does not seem to do a good job: the estimate of the string risk premium is not significant and, sometimes, comes with a counterintuitive negative sign. In fact, a negative sign of ϕ(⋅,j) for some asset *j* may turn out to be an interesting property, as discussed below; however, Model I estimates imply that, for certain test portfolios, $\phi (\cdot ,j)=\bar{\phi }$ is negative, implying that the unit risk-premium is negative *for all* *j*. Furthermore, for half of the test portfolios, there is an insignificant relation between predicted and realized returns. Finally, the unconditional pricing performance is quite poor, with ${\bar{R}}^{2}$ ranging from negative to less than 20%. Allowing for variation in the premium in the cross-sectional dimension turns out to be very important, producing significant parameter estimates of $\phi_{0}$ and $\phi_{1}$. For all the test portfolios, the model has a reasonable pricing fit, with cross-sectional ${\bar{R}}^{2}$ varying from 20% to nearly 80%, with the best fit displaying at the level of the global ME-OP portfolios. Time-variation in the string risk premium (Model III) provides a marginally improved performance (see table 6), with results very similar to those of Model II. Figure 6 provides scatterplots of the unconditional expected returns predicted by Model III against average realized returns.

**Figure 7.**
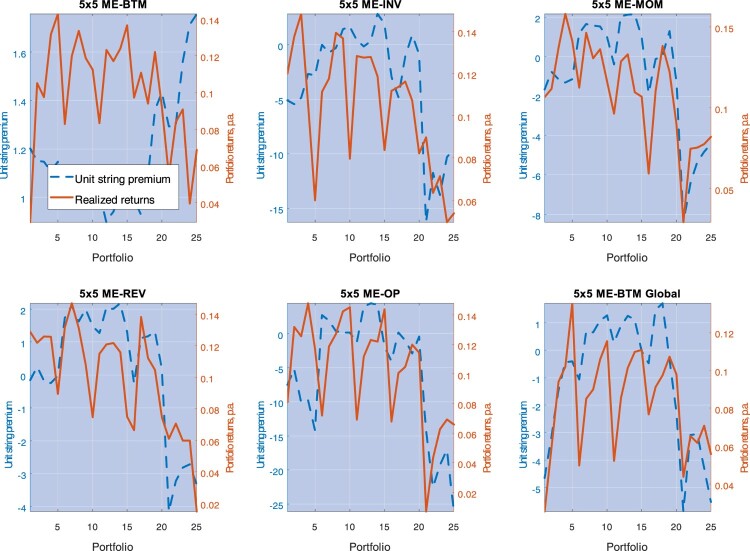
This picture depicts average excess returns and the unit risk premia for each portfolio, with the latter estimated from Model III. The estimates are performed for 5×5 ME-BTM, 5×5 ME-INV, 5×5 ME-MOM, 5×5 ME-REV, 5×5 ME-OP, and 5×5 ME-BTM Global portfolios.

Which portfolios contribute to the unconditional premia displayed in figure 6? What are the model predictions on the correlation premia conditional on the realization of $y_{t}$ (say, $C(y_{t},i)$ in equation (16))? Figure 7 plots average unit string premia, $\phi (\bar{y},j)$, for each portfolio test, across all portfolios *j* comprising that test, obtained while fixing the state variable at the sample value taken by $\bar{y}=-\ln{\bar{Y}}_{\left(1\right)}$ over the sample size. In each test, the first 5 portfolios correspond to those with the smallest size (i.e. in the first quantile) and are ordered from the lowest to the highest characteristics (for example, in the case of the 5×5 ME-OP portfolio test, from the lowest to the highest operating profitability); portfolios from 5*i* + 1 through 5(i+1) are ordered similarly, with *i* = 1 identifying portfolios with the second smallest size quantile, and *i* = 4 identifying portfolios with the biggest size. The picture also depicts average returns on all portfolios.

The unit string premia track the ‘shark-tooth’ pattern of the portfolios' expected returns quite well: with the exception of the first portfolio test (5×5 BE-BTM), the higher the average return on portfolio-*j*, the higher $\phi (\bar{y},j)$ in general. Furthermore, $\phi (\bar{y},j)$ is negative for both small and big size portfolios. To facilitate the interpretation of these findings, recall the heuristic explanations in table 1. The contribution of portfolio-*j* to the premium of portfolio-*i* is proportional to $\phi (y_{t},j)\rho (y_{t},i,j)$, where $\rho (y_{t},i,j)$ is the risk of co-variation that portfolio-*i* returns have with *j*, and $\phi (y_{t},j)$ is the unit risk premium commanded by any asset for the exposure to portfolio-*j* returns. Thus, portfolios with negative $\phi (\bar{y},j)$ may be interpreted as ‘correlation-hedges’, in that assets more exposed to them require lower overall expected returns. Figure 7 shows very clearly that *small* size and *big* size portfolios are such correlation hedges. However, exposure to *middle* size portfolios commands positive unit string premia. We shall return to the correlation-hedge properties of big size portfolios in a moment.

We qualify our findings: How do conditional premia relate to the average market correlation? The key relation is that between the unit string premium and $\varrho_{q}(j)$, q∈{1,2}, the portfolios' exposures to the average market correlation (see Section 3.2):

$$\phi \left(y_{t},j\right)=\phi_{v0}\varrho_{0}\left(j\right)+\left(\phi_{v1}+\phi_{v2}e^{-y_{t}}\right)\varrho_{1}\left(j\right).$$

These two exposures have a natural interpretation. Note that the dynamic GCE introduced in (14),

(29) $$\begin{array}{rl} \rho \left(y_{t},j\right) & \equiv \int_{0}^{1}\rho \left(y_{t},i,j\right)di\\ & =\;\underset{\mathrm{constant}\;\mathrm{connectivity}}{\underbrace{\varrho_{0}\left(j\right)}}+\underset{\mathrm{conditional}\;\mathrm{connectivity}}{\underbrace{\varrho_{1}\left(j\right)e^{-y_{t}}}}, \end{array}$$

is a measure of total connectivity of portfolio-*j* to all remaining assets in a given test. Now, the parameter estimates in Table 6 suggest that $\phi_{v0}>0$, that is, the unit string premium $\phi (y_{t},j)$ for portfolio-*j* increases with the constant connectivity component of portfolio-*j*, $\varrho_{0}(j)$. The only exception is the first portfolio test, which, from now on, we shall not comment on.

**Figure 8.**
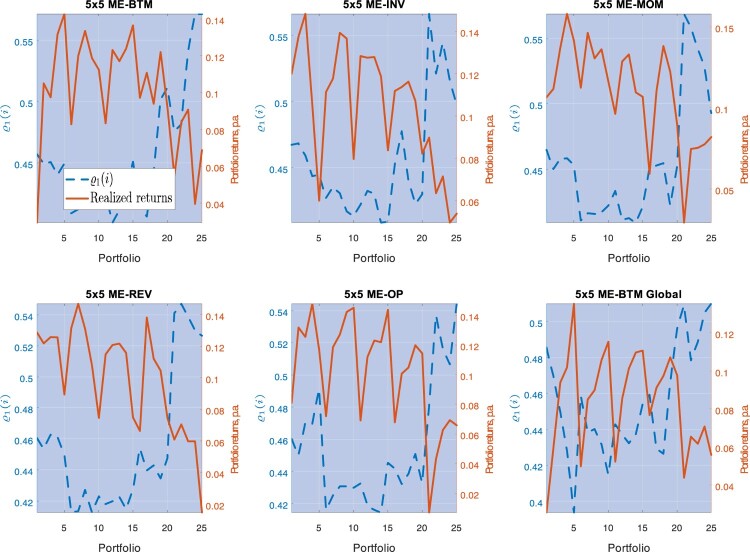
This picture depicts average excess returns and the sensitivity of the conditional sensitivity, $\varrho_{1}(j)$, for each portfolio, with the latter estimated from Model III. The estimates are performed for 5×5 ME-BTM, 5×5 ME-INV, 5×5 ME-MOM, 5×5 ME-REV, 5×5 ME-OP, and 5×5 ME-BTM Global portfolios.

More subtle is the relation between the unit string premium and the variable part of $\rho (y_{t},j)$ in (29), i.e. the *conditional* connectivity of portfolio-*j*. Note that $\varrho_{1}(j)$ measures the sensitivity of portfolio-*j* total connectivity to changes in $y_{t}$. Table 6 estimates suggest that $\phi_{v1}+\phi_{v2}e^{-{\hat{y}}_{t}}<0$ for all ${\hat{y}}_{t}$, such that the unit string premium $\phi ({\hat{y}}_{t},j)$ decreases with $\varrho_{1}(j)$ for all of our test portfolios. That is, portfolios with higher $\varrho_{1}(j)$ provide better correlation hedges. Figure 8 shows that portfolios with the highest $\varrho_{1}(j)$ tend to be *big* size, and that these portfolios also command lower average returns. Note that these properties are specific to big stocks: in Appendix 3, we find that the unit string premium generally *increases* with $\varrho_{1}(j)$ for S&P 500 sectors and index-based portfolios. Furthermore, note that for some of the test portfolios in Table 6, $\phi_{v2}<0$; in these cases, the previous effects become stronger in bad times: the lower $y_{t}$, the stronger the inverse relation between the unit string premium and conditional connectivity. Now, big stocks provide ‘dynamic correlation-hedges’: after a negative shock in $y_{t}$, assets that are more exposed to stocks with higher conditional connectivity (i.e. assets *i* with a higher $\rho (y_{t},i,j)$) require a lower premium in bad times.

**Figure 9.**
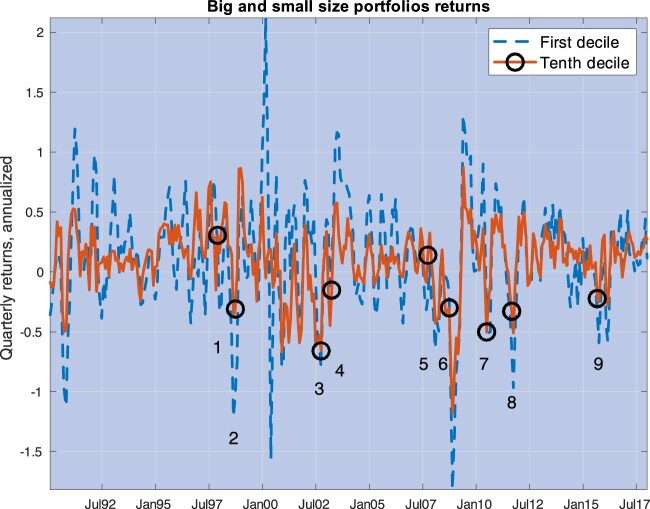
This picture depicts quarterly realized returns of portfolios sorted by size in the first decile (dashed, blue line) and the tenth decile (‘big size’) (dashed, blue line). The numbered circles identify the same events in figure 5 (see table 3) and are placed around the realization of big size portfolios returns.

These correlation-hedge properties suggest one interpretation of the low average returns that big stocks display: big stocks provide some hedging properties, which manifest through the previous correlation-hedge channel; investors seek exposure to them, and these stocks, then, provide low average returns. It remains an open question as to why big stocks' connectivity increases in bad times, and why investors seek exposure to them–that is, why big stocks have such hedging properties. A natural explanation is that big stocks are likely to be resilient to systemic shocks (i.e. shocks by which market correlations become high), and that these stocks are also the most interconnected with the rest of the economy. Consistent with this hypothesis, we find that big size portfolios are more resilient than small during systemic events, and quite substantially. Precisely, we calculate quarterly returns for portfolios in the first (small) and tenth (big) decile and find that, while these returns average 12.72% and 10.13%, respectively, big size portfolios realized (annualized) returns are on average 12.40% *higher* than small across the nine systemic events identified in figure 5 (see table 3). Figure 9 depicts these returns in our sample, along with these systemic events.

**Figure 10.**
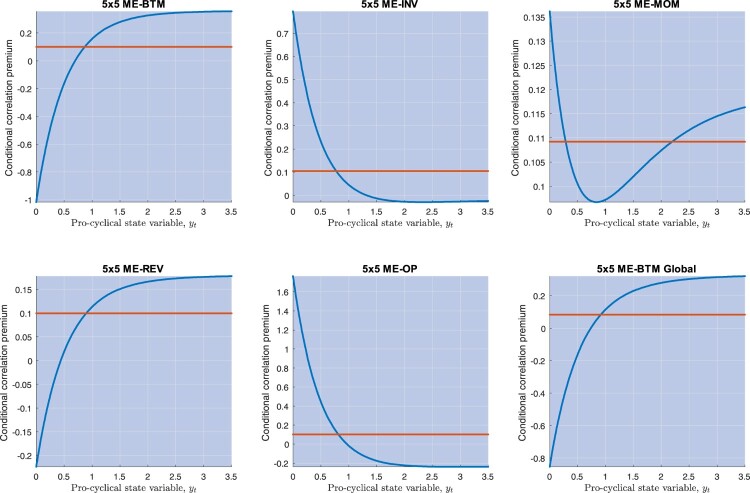
This picture depicts the relation between the conditional correlation premium predicted by Model III (equally weighted across all portfolios) and the state variable $y_{t}$ regarding 5×5 ME-BTM, 5×5 ME-INV, 5×5 ME-MOM, 5×5 ME-REV, 5×5 ME-OP, and 5×5 ME-BTM Global portfolios. The red, horizontal line is the unconditional premium for each of these portfolios.

When $\phi_{v2}<0$, assets that are more exposed to big stocks even require a lower premium in bad times. These properties imply that some test portfolios, i.e. those for which there is a sufficiently high exposure to big size portfolios, may exhibit a cross-section average premium that is pro-cyclical: their expected returns decrease in times of high market correlations. Figure 10 shows that this is the case of the 5×5 ME-REV and 5×5 ME-BTM Global test portfolios. Figure 10 also shows that, in the remaining cases, expected returns may be both counter-cyclical (5×5 ME-INV and 5×5 ME-OP) or non-monotonic in $y_{t}$ (5×5 ME-MOM).

### 4.4. Premium for correlation risk

Next, we examine the model predictions on the premium for correlation risk. Proposition 3.3 (see Section 3) suggests a theoretical relation between realized correlations and the premium for correlation risk. In Section 3 we explained that, given our parameter estimates, this relation should be roughly inverse for most of the time (see figure 4). We calculate data counterparts to this relation. We approximate the premium for correlation risk with its *realized* counterpart, defined as the difference between average correlations (implied and historical) over the past 22 days. We also compute the model-implied realized premium for correlation risk, estimating *P*-correlations through the average correlations $\rho (y_{t};i,j)$ calculated over the last 22 days, and relying on the estimates of the state $y_{t}$ in Section 4.2.

**Figure 11.**
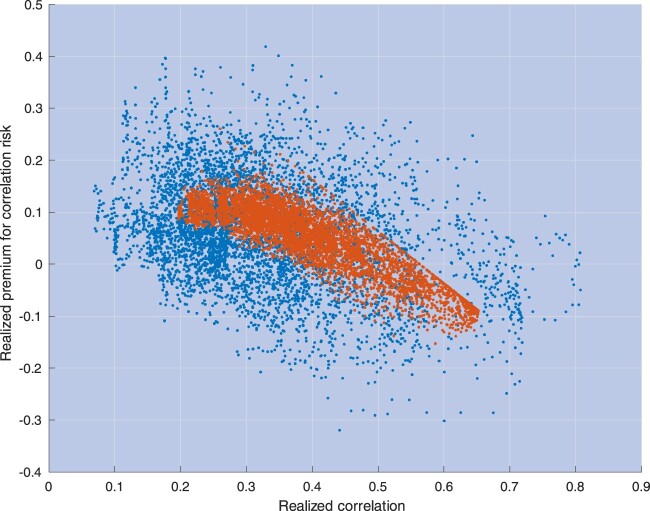
This picture depicts scatterplots of realized premium for correlation risk for S&P 500 stocks against one-month realized correlations. Blue and red dots identify pairs in the data and pairs predicted by the model, respectively.

Figure 11 plots the results. The model predicts that the premium for correlation risk is statistically inversely related to realized correlations, as in the data. In terms of the explanations of Proposition 3.3 in Section 3, in bad times, when implied and realized correlations are both high, the premium for correlation risk decreases: implied correlations are obviously bounded and, then, a further increase in both correlations may translate into a decreasing difference between implied and realized correlations. Figure 11 shows that this effect is so strong that the premium for correlation risk is negatively related to realized correlations.

**Figure 12.**
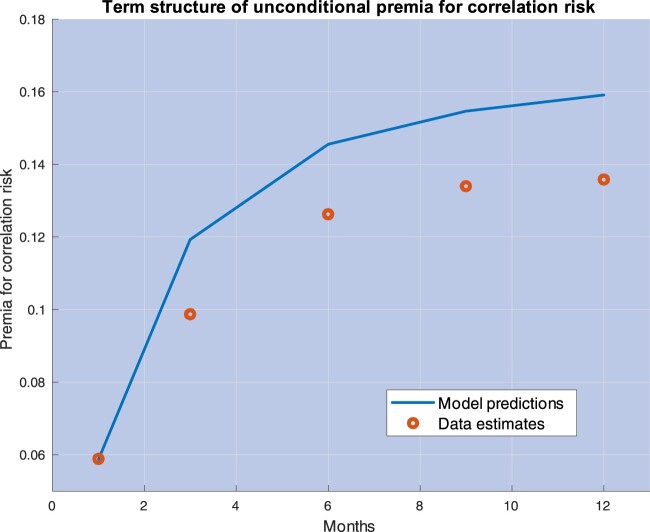
This picture depicts the unconditional premium for correlation risk calculated for horizons equal to 1, 3, 6, 9, and 12 months. The circles are data estimates, computed as described in the main text. The solid curve depicts model predictions, obtained while fixing parameter values at the GMM estimates in (27), which rely on one moment condition based on one-month unconditional premium.

Because implied correlations are on average higher than realized, we might, then, also expect that implied correlations move less than one-to-one with realized correlations. It is indeed the case. Table 8 reports regression estimates that reveal this property holds both in the data and for the model. These properties are in contrast with the empirical evidence in the equity volatility space, where volatility risk-premia do actually increase in bad times (see Corradi *et al.* 2013).^8^

**Table 8.** This table provides estimates (with standard errors in parenthesis) and adjusted- $R^{2}$ for the coefficients *a* and *b* in the linear regression $\rho_{Q}=a+b\rho_{P}$, where $\rho_{Q}$ is the one-month expected correlation for S&P 500 stocks under the risk-neutral probability, *Q*, and $\rho_{P}$ is the one-month realized correlation.

	*a*	*b*	${\bar{R}}^{2}$
Data	(0.0034)	(0.0096)	46%
	0.1804	0.6337	
Model	(0.0015)	(0.0038)	76%
	0.2508	0.4913	

Finally, we examine the model implications on the term structure of unconditional premia for correlation risk. The GMM estimator in (27) relies on moment conditions that include the unconditional *one-month* premium for correlation risk. Yet our model allows us to consider any arbitrary horizon. Figure 12 plots the average premium for correlation risk estimated from data along with the expression for $E(P(y_{t};\vartheta ))$ in (26), calculated with parameter values based on our GMM estimates. The model reproduces the upward sloping curve in the data and comes close to quantitatively match the unconditional premia for correlation risk estimated on data at all horizons up to one year.

## 5. Conclusion

This paper introduces an arbitrage pricing model by which the cross-section of expected returns relates to the granular exposure of each asset return with respect to all remaining returns. That is, we model asset risk premia as being directly driven by the very same assets' correlations, and not by a number of a pre-determined factors. More precisely, our model takes asset returns to be driven by the realization of a string, which, then, determines returns co-movements and the whole set of correlations amongst asset returns. In this setup, ‘risk’ is, thus, determined by the joint fluctuations of asset returns in a given universe of securities, and the cross-section of expected returns reflects the exposures of any given asset price fluctuations to the fluctuations of the remaining asset prices. The cross-section of expected returns is simply given by these exposures, weighted through a premium functional.

Within this theoretical framework, we specify a number of models that we use in empirical work. We assume that the assets correlations in the string are random. While our econometric methodology only requires asset returns to estimate the cross-section of expected returns, we also use the cross-section of options on individual S&P500 components and extract information on the unobserved state underlying realized correlations at any given point in time. We develop method-of-moments conditions that we employ to estimate our model. With our estimates of the state, we reconstruct the dynamics of average correlations and premium for correlation risk, and, naturally, the cross-section of expected returns that are predicted by the model.

The model predicts the empirical patterns of premia for correlation risk, but also explains cross-sectional pricing in a number of portfolios, both in the U.S. and in the international stock universe, with a performance that is often better than that of standard linear factor models. The model predictions shed new light into the empirical properties of big shocks. Big stocks are correlation-hedges, in that assets that are more exposed to them require lower expected returns. Under conditions, portfolios particularly exposed to big stocks may even require lower returns in bad times (when all assets' correlations spike) than in good. The string model and its granular methodology provide a flexible and a complementary framework to the standard factor structure that may be used for cross-sectional asset pricing and also for quantifying risks that any individual portfolio may have in common with the whole cross-section of asset returns.

## Data Availability

The authors confirm that the data supporting the findings of this study are available within its supplementary materials (available upon request).

## Footnotes

1 If Brownian motions can be thought of as particles that move randomly over time, a two-parameter random field can be thought of as the random motion of a string. A three-parameter random field is also known as a membrane. This paper deals with strings.

2 Thus, we rely on a factor model for *asset returns correlations*. The point of the paper is that we do not rely on a factor model for the cross-section of *asset returns*.

3 The premium for correlation risk compensates for the risk that correlations are random. Furthermore, in this paper, we are referring to the cross-section of expected returns (our main focus) as correlation premia to emphasize the role correlation plays within our string model. As noted earlier, our model predicts correlation premia could be non-zero even if correlations are constant (in which case the premium for correlation risk would be zero).

4 Numerous studies have devoted their attention to size characteristics and variations of the related factor. To name just a few, both Chan *et al.* (1985), and Chan and Chen (1991) looked at characteristics of small and large firms and the firm size effect; Fama and French (1992) analyzed the cross-section of stock returns versus characteristics; Fama and French (1995) connected size effects to the properties of firms' earnings. Later studies such as Campbell *et al.* (2008) more specifically focussed on rationalizing size effects by connecting them to financial distress risk.

5 One instance of such an inverse relation is documented by Mueller *et al.* (2017) in the foreign exchange market.

6 Elton and Gruber (1973) are amongst the first to suggest this notion of correlation under the physical probability. Driessen *et al.* (2005) and Skinzi and Refenes (2005) extended this notion to the risk-neutral space to measure an average option-implied correlation representative of a universe of stocks.

7 Precisely, $\bar{P}({\hat{\vartheta }}_{N})$ is the one-month realized average of $P(y_{t};{\hat{\vartheta }}_{N})$ in (21) evaluated at the estimated parameter vector ${\hat{\vartheta }}_{N}$.

8 Corradi *et al.* (2013) (Section 4.2.5) provide such evidence relying on ex-ante volatility risk-premia, within a no-arbitrage model for equity volatility. In the interest rate volatility space, Mele *et al.* (2015) and Mele and Obayashi (2015) study some properties of the volatility risk-premium, without addressing the issue of the premium sensitivity to market conditions.

## Appendix 1. Proofs

Proof (Proof of Proposition 3.2) Consider, first, the following preliminary result: for any given ℓ,

(A1)
$$E_{t}\left(e^{-\ell y_{T}}\right)={\bar{a}}_{\ell }\left(T-t\right)e^{-{\bar{b}}_{\ell }\left(T-t\right)\ell y_{t}},$$

where

$$\begin{array}{rl} & {\bar{a}}_{\ell }\left(x\right)={\left(\frac{2\kappa }{2\kappa +\ell \eta^{2}\left(1-e^{-\mathrm{\kappa x}}\right)}\right)}^{\frac{2\mathrm{\kappa m}}{\eta^{2}}},\\ & {\bar{b}}_{\ell }\left(x\right)=\frac{2\kappa e^{-\mathrm{\kappa x}}}{2\kappa +\ell \eta^{2}\left(1-e^{-\mathrm{\kappa x}}\right)}. \end{array}$$

Equation (A1) follows by a mere change in notation in a result to be stated below (see equation (A5)). Taking the limits leaves

$$E\left(e^{-\ell y_{t}}\right)=\lim_{T\to \infty }E_{t}\left(e^{-\ell y_{T}}\right)={\bar{Y}}_{\left(\ell \right)},$$

where ${\bar{Y}}_{\left(\ell \right)}$ is defined in the proposition. The expressions for the unconditional expected returns in Proposition 3.2 immediately follow.

Before providing the proof of Proposition 3.3, we prove a statement given in the main text regarding the dynamics of the state variable $y_{t}$ under the risk-neutral probability.

Proposition A.1 (Dynamics of *y* under *Q*) Consider two diffusion processes, $x_{\mathrm{it}}$, *i* = 1, 2, solutions to equation (20), viz

$$dx_{\mathrm{it}}=\left(\mathrm{\kappa m}-\left(\kappa +\nu_{i}\eta \right)x_{\mathrm{it}}\right)dt+\eta \sqrt{x_{\mathrm{it}}}\;d{\tilde{W}}_{t},$$

where $\nu_{1}>\nu_{2}$. Then, $x_{1t}\leq x_{2t}$ a.s.

Proof. The drift of $x_{1t}$ is strictly less than the drift of $x_{2t}$, and the proposition follows by a comparison theorem (e.g. Karatzas and Shreve (1991, pp. 291–295)).

Proof of Proposition 3.3. We provide details regarding the function $w(y_{t},T-t)\equiv u(y_{t},\tau -t;\theta ,0)=E_{t}(e^{-y_{T}})$ in equation (22), as those regarding $E_{t}^{Q}(e^{-y_{T}})$ follow through a change in notation. The function w(y,T−τ) satisfies the following partial differential equation

$$\begin{array}{rl} 0 & =-w_{2}\left(y,T-\tau \right)+\kappa \left(m-y\right)w_{1}\left(y,T-\tau \right)\\ & \;+\frac{1}{2}\eta^{2}yw_{11}\left(y,T-\tau \right),\;\mathrm{for}\;\mathrm{all}\;\tau \in [t,T), \end{array}$$

where subscripts denote partial derivatives. The boundary condition is $w(y,0)=e^{-y}$. Conjecture that $w(y_{t},T-t)=e^{\alpha (T-t)-b(T-t)y_{t}}$ and plug this suggested function into the previous partial differential equation. The result is that *α* and *b* satisfy the following ordinary differential equations: for all x∈(0,T−t],

$$\left\{ \begin{array}{l} 0=\dot{b}\left(x\right)+\mathrm{\kappa b}\left(x\right)+\frac{1}{2}\eta^{2}b^{2}\left(x\right)\\ 0=\dot{\alpha }\left(x\right)+\mathrm{\kappa mb}\left(x\right) \end{array}\right.$$

subject to the boundary conditions α(0)=0 and b(0)=1. The solution for *b* and *α* follow by standard integration arguments and details are available upon request. Equation (22) and, then, equation (21) follow by taking the exponential, $a=e^{\alpha }$, and noting that $\mathrm{\kappa m}=\tilde{\kappa }\tilde{m}$. Next, we show that, for ν>0, P is (i) strictly positive, (ii) increasing and concave in *y* for low *y*, and (iii) decreasing and convex in *y* for high *y*. (Note, also that the arguments below would equally go through if $\varrho_{1}<0$ and ν<0.) The first property directly follows by Proposition A.1. However, we provide an alternative proof based on an argument that will be used to deal with the other proofs of the proposition. Note that the function Δu(y,T−t)≡u(y,τ−t;θ,ν)−u(y,τ−t;θ,0) is solution to the following partial differential equation

(A2)
$$0=L\Delta u\left(y,T-\tau \right)-\mathrm{\nu \eta y}u_{1}\left(y,T-\tau \right),\;\mathrm{for}\;\mathrm{all}\;\tau \in [t,T),$$

where $Lf(y,T-t)=\frac{\partial }{\partial t}f(y,T-t)+\kappa (m-y)\frac{\partial }{\partial y}f(y,T-t)+\frac{1}{2}\eta^{2}y\frac{\partial^{2}}{\partial y^{2}}f(y,T-t)$, and subject to the boundary condition Δu(y,0)=0. Therefore, by the maximum principle for partial differential equations, we have that the sign of Δu(y,T−t) is the same as the sign of $-\mathrm{\nu \eta y}u_{1}(y,\tau -t)$. Since u(y,τ−t) is strictly decreasing in *y* for any finite *T*, it follows that Δu(y,T−t) is strictly positive, and so is P. Regarding the second property (increasing and concave for low *y*) and the third (decreasing and convex for high *y*), differentiate equation (A2) two times with respect to *y*, and denote with $\Delta u_{1}(y,T-t)$ and $\Delta u_{11}(y,T-t)$ the first and the second partial of Δu(y,T−t) with respect to *y*. The result is that $\Delta u_{1}(y,T-t)$ and $\Delta u_{11}(y,T-t)$ are solutions to the following partial differential equations

(A3)
$$\begin{array}{rl} 0 & =L\Delta u_{1}\left(y,T-\tau \right)\\ & \;+\mathrm{\nu \eta b}\left(T-\tau \right)u\left(y,T-\tau \right)\left(1-b\left(T-\tau \right)y\right),\;\mathrm{for}\;\mathrm{all}\;\tau \in [t,T), \end{array}$$

and

(A4)
$$\begin{array}{rl} 0 & =L\Delta u_{11}\left(y,T-\tau \right)\\ & \;-\nu \eta b^{2}\left(T-\tau \right)u\left(y,T-\tau \right)\left(2-b\left(T-\tau \right)y\right),\;\mathrm{for}\;\mathrm{all}\;\tau \in [t,T), \end{array}$$

subject to the boundary conditions $\Delta u_{1}(y,0)=0$ and $\Delta u_{11}(y,0)=0$. Equation (A3) can be rearranged to yield

$$\begin{array}{rl} & \Delta u_{1}\left(y_{t},T-t\right)\\ & =\nu \eta \int_{t}^{T}b\left(T-\tau \right)E_{t}\left[u\left(y_{\tau },T-\tau \right)\left(1-b\left(T-\tau \right)\right)y_{\tau }\right]d\tau \\ & =\nu \eta E_{t}\left[u\left(y_{\tau },T-\tau \right)\right]\int_{t}^{T}b\left(T-\tau \right)E_{t}^{*}\left[1-b\left(T-\tau \right)y_{\tau }\right]d\tau \\ & =\nu \eta E_{t}\left[u\left(y_{\tau },T-\tau \right)\right]\int_{t}^{T}b\left(T-\tau \right)\left[1-b\left(T-\tau \right)E_{t}^{*}\left(y_{\tau }\right)\right]d\tau , \end{array}$$

where $E_{t}^{*}(\cdot )$ denotes the expectation is taken under the probability $P^{*}$, defined as

$${\frac{dP^{*}}{dP}|}_{F_{\tau }}=\frac{u\left(y_{\tau },T-\tau \right)}{E_{t}\left[u\left(y_{\tau },T-\tau \right)\right]}.$$

By the no-crossing property of a diffusion, the expectation $E_{t}^{*}(y_{\tau })$ is increasing in the initial condition $y_{t}$ and, thus, there exists a threshold $y_{A}$ (resp., $y_{B}$) such that for all $y_{t}<y_{A}$ (resp., $y_{t}>y_{B}$), $\Delta u_{1}(y_{t},T-t)$ is positive (resp., negative). Based on equation (A4), we can make a similar argument and conclude that there exists a threshold $y_{C}$ (resp., $y_{D}$) such that for all $y_{t}<y_{C}$ (resp., $y_{t}>y_{D}$), $\Delta u_{11}(y_{t},T-t)$ is negative (resp., positive).

Proof of Proposition 4.1. The *n*-th conditional moment of $\rho (y_{T};\varrho )$ is

Et(ρn(yT;ϱ))=Et(ϱ0+ϱ1e−yT)n=Et(∑i=0n(ni)ϱ0iϱ1n−ie−(n−i)yT)=∑i=0n(ni)ϱ0iϱ1n−iEt(e−(n−i)yT),

where the second line follows by the binomial formula. Now, by Itô's lemma, $z_{i,t}\equiv (n-i)y_{t}$ is solution to

$$dz_{i,t}=\kappa \left(m_{i}-z_{i,t}\right)dt+\eta_{i}\sqrt{z_{i,t}}\;dW_{t},$$

where $m_{i}=(n-i)m$ and $\eta_{i}=\sqrt{n-i}\eta$. Therefore, by the expression for the conditional expectation of $e^{-y_{T}}$ in Proposition 4.1,

(A5)
$$E_{t}\left(e^{-\left(n-i\right)y_{T}}\right)=a_{i}\left(T-t\right)e^{-b_{i}\left(T-t\right)\left(n-i\right)y_{t}},$$

where, and using the fact that $m_{i}/\eta_{i}^{2}=m/\eta^{2}$,

$$\begin{array}{rl} a_{i}\left(x\right) & ={\left(\frac{2\kappa }{2\kappa +\eta_{i}^{2}\left(1-e^{-\mathrm{\kappa x}}\right)}\right)}^{\frac{2\mathrm{\kappa m}}{\eta^{2}}},\\ b_{i}\left(x\right) & =\frac{2\kappa e^{-\mathrm{\kappa x}}}{2\kappa +\eta_{i}^{2}\left(1-e^{-\mathrm{\kappa x}}\right)}. \end{array}$$

Equation (24) follows by taking the limit $E(\rho^{n}(y_{t};\varrho ))=\lim_{T\to \infty }E_{t}(\rho^{n}(y_{T};\varrho ))$. Next, we determine the following unconditional uncentered covariance

(A6)
$$c\rho_{\infty }^{\Delta }\equiv \lim_{T\to \infty }E_{t}\left(\rho \left(y_{T};\varrho \right)\rho \left(y_{T+\Delta };\varrho \right)\right).$$

We have

$$\begin{array}{rl} & E_{t}\left(\rho \left(y_{T};\varrho \right)\rho \left(y_{T+\Delta };\varrho \right)\right)\\ & =\varrho_{0}^{2}+\varrho_{0}\varrho_{1}\left(E_{t}\left(e^{-y_{T}}\right)+E_{t}\left(e^{-y_{T+\Delta }}\right)\right)+\varrho_{1}^{2}E_{t}\left(e^{-\left(y_{T}+y_{T+\Delta }\right)}\right). \end{array}$$

By the Law of Iterated Expectations, and the expression for the conditional expectation of $e^{-y_{T}}$ in Proposition 3.3,

$$E_{t}\left(e^{-\left(y_{T}+y_{T+\Delta }\right)}\right)=E_{t}\left(e^{-y_{T}}E_{T}\left(e^{-y_{T+\Delta }}\right)\right)=a_{\Delta }E_{t}\left(e^{-\left(1+b_{\Delta }\right)y_{T}}\right),$$

where $a_{\Delta }=a(\Delta ;0)$ and $b_{\Delta }=b(\Delta ;0)$ and a(x;ν) and a(x;ν) are as in equation (22) of Proposition 3.3. Applying again the expression for the conditional expectation of $e^{-y_{T+\Delta }}$ in Proposition 4.1 and relying on arguments nearly identical to those used to derive the conditional moment in equation (A5),

$$E_{t}\left(e^{-\left(1+b_{\Delta }\right)y_{T}}\right)=a^{\Delta }\left(T-t\right)e^{-b^{\Delta }\left(T-t\right)\left(1+b_{\Delta }\right)y_{t}},$$

where

$$\begin{array}{rl} a^{\Delta }\left(x\right) & ={\left(\frac{2\kappa }{2\kappa +\left(1+b_{\Delta }\right)\eta^{2}\left(1-e^{-\mathrm{\kappa x}}\right)}\right)}^{\frac{2\mathrm{\kappa m}}{\eta^{2}}},\\ b^{\Delta }\left(x\right) & =\frac{2\kappa e^{-\mathrm{\kappa x}}}{2\kappa +\left(1+b_{\Delta }\right)\eta^{2}\left(1-e^{-\mathrm{\kappa x}}\right)}. \end{array}$$

Hence,

$$E_{t}\left(e^{-\left(y_{T}+y_{T+\Delta }\right)}\right)=a_{\Delta }a^{\Delta }\left(T-t\right)e^{-b^{\Delta }\left(T-t\right)\left(1+b_{\Delta }\right)y_{t}}.$$

Therefore, the limit in (A6) is obtained as

$$\begin{array}{rl} c\rho_{\infty }^{\Delta } & =\varrho_{0}^{2}+2\varrho_{0}\varrho_{1}\lim_{x\to \infty }a\left(x;\nu \right)+\varrho_{1}^{2}a_{\Delta }\lim_{x\to \infty }a^{\Delta }\left(x\right)\\ & =\varrho_{0}^{2}+2\varrho_{0}\varrho_{1}{\left(\frac{2\kappa }{2\kappa +\eta^{2}}\right)}^{\frac{2\mathrm{\kappa m}}{\eta^{2}}}\\ & \;+\varrho_{1}^{2}{\left(\frac{4\kappa^{2}}{4\kappa^{2}+4\kappa \eta^{2}+\eta^{4}\left(1-e^{-\kappa \Delta }\right)}\right)}^{\frac{2\mathrm{\kappa m}}{\eta^{2}}}. \end{array}$$

Equation (25) follows by rearranging terms in

$$\mathrm{cov}\left(\rho \left(y_{t};\varrho \right),\rho \left(y_{t+\Delta };\varrho \right)\right)=c\rho_{\infty }^{\Delta }-E{\left(\rho \left(y_{t};\varrho \right)\right)}^{2},$$

where the expression for $E(\rho (y_{t};\varrho ))$ is obtained through equation (24) of the proposition.

Proof Proof of equation (26) We have, for *l*>*t*, and for fixed Δt≡T−t,

(A7)
$$\begin{array}{rl} E\left(P\left(y_{t};\vartheta \right)\right) & =\lim_{l\to \infty }E_{t}\left(P\left(y_{l};\vartheta \right)\right)\\ & =\frac{\varrho_{1}}{\Delta t}\int_{0}^{\Delta t}\lim_{l\to \infty }E_{t}\left(u\left(y_{l},x;\theta ,\nu \right)-u\left(y_{l},x;\theta ,0\right)\right)dx. \end{array}$$

By Proposition 4.1, and arguments similar to those leading to equation (A5),

$$\begin{array}{rl} E_{t}\left(u\left(y_{l},x;\theta ,\nu \right)\right) & =a\left(x;\nu \right)E_{t}\left(e^{-b\left(x;\nu \right)y_{l}}\right)\\ & =a\left(x;\nu \right)a_{B}\left(l-t;\nu \right)e^{-b_{B}\left(l-t;\nu \right)b\left(x;\nu \right)y_{t}}, \end{array}$$

where

$$\begin{array}{rl} & a_{B}\left(l-t;\nu \right)\equiv {\left(\frac{2\kappa }{2\kappa +b\left(x;\nu \right)\eta^{2}\left(1-e^{-\kappa \left(l-t\right)}\right)}\right)}^{\frac{2\mathrm{\kappa m}}{\eta^{2}}},\\ & b_{B}\left(l-t;\nu \right)\equiv \frac{2\kappa e^{-\kappa \left(l-t\right)}}{2\kappa +b\left(x;\nu \right)\eta^{2}\left(1-e^{-\kappa \left(l-t\right)}\right)}. \end{array}$$

Equation (26) follows by calculating the limits in (A7), using the definition of a(x;ν) and b(x;ν) in Proposition 4.1, and rearranging terms.

## Appendix 2. Extensions

### A.1. A string-and-factor model of asset returns

We extend the model in Section 2 to a market in which asset returns are strings, but they are also affected by systematic factors driven by Brownian motions, assuming that

(A8)
$$\begin{array}{rl} & \frac{dP_{t}\left(i\right)+D_{t}\left(i\right)dt}{P_{t}\left(i\right)}\\ & =E\left(y_{t},i\right)dt+\sigma \left(y_{t},i\right)dZ_{t}\left(i\right)+v\left(y_{t},i\right)dW_{t},\;i\in \left(0,1\right), \end{array}$$

where $W_{t}$ is a standard multidimensional Brownian motion, v(y,i), is a continuous function, in y and *i*, and represents the asset returns exposures to the systematic factors, the ‘betas.’ Remaining notation is as in equation (1).

The pricing kernel is still as in equation (5), such that repeating the arguments leading to Proposition 3.3, but relying on equation (A8), leaves the following expression for the expected excess returns on any asset- i∈(0,1)

(A9)
$$E\left(y_{t},i\right)-r\left(y_{t}\right)=C\left(y_{t},i\right)+v\left(y_{t},i\right)\lambda \left(y_{t}\right),$$

where C(y,i) is as in equation (9). Compared to Proposition 2.1, this formulation adds a standard factor-risk premium to the explanation of the cross-section of asset returns, v(y,i)λ(y).

### A.2. Compound strings

We consider the following extension to equation (1)

(A10)
$$\begin{array}{rl} & \frac{dP_{t}\left(i\right)+D_{t}\left(i\right)dt}{P_{t}\left(i\right)}\\ & =E_{t}\left(y_{t},i\right)dt+\sigma \left(y_{t},i\right)dZ_{t}\left(i\right)+w\left(y_{t},i\right)dZ_{t}\left(Z,y_{t}\right), \end{array}$$

where

$$dZ_{t}\left(Z,y_{t}\right)=\int_{0}^{1}n\left(y_{t},j\right)dZ_{t}\left(j\right)dj,$$

for some functions $w(y_{t},i)$ and $n(y_{t},i)$. The additional term, $dZ_{t}(Z,y_{t})$, is a linear functional of the whole string, and will be referred to as *compound string* in the sequel. Note that the state, $y_{t}$, may not necessarily be a diffusion process. It may be a string, as in the example developed in Appendix A.3.

This extension accounts for economies in which each asset return reacts to shocks in the fundamentals pertaining to all remaining asset returns, that is, not only to ‘its own point-*i*’ of the string $dZ_{t}(i)$, but also to $dZ_{t}(j)$ for all *j*, directly. For example, in the illustrative model of Appendix A.3 , each asset return is driven by a shock on its dividend and, due to market clearing, on those affecting all the dividend shares (i.e. the proportions of aggregate dividends paid by each asset), leading to price dynamics that are a special case of equation (A10).

By arguments similar to those leading to Proposition 2.1, the expected excess returns on each asset are now given by

(A11)
$$\begin{array}{rl} & E_{t}\left(y_{t},i\right)-r\left(y_{t}\right)\\ & =\sigma \left(y_{t},i\right)\int_{0}^{1}\phi \left(y_{t},j\right)\rho \left(y_{t},i,j\right)dj\\ & \;+w\left(y_{t},i\right)\iint_{u,v\in {\left[0,1\right]}^{2}}\phi \left(y_{t},u\right)n\left(y_{t},v\right)\rho \left(y_{t},u,v\right)du\;dv. \end{array}$$

The first term on the R.H.S. of equation (A11) is the expected return predicted by Proposition 2.1. The second term captures the premium due to the compound string in equation (A10). In our empirical work, we rely on the simple specification of the model that gives rise to Proposition 2.1. However, we now provide an example of a Consumption-based CAPM that leads to the assumptions underlying the predictions of equation (A11).

### A.3. Example: A consumption-based CAPM

We consider a string version of a multi-asset economy in Mele (2022, Section 9.9.1, p. 499), that is, an infinite horizon economy with a continuum of long-lived securities in i∈(0,1). Each of these securities delivers an instantaneous dividend $D_{t}(i)$ at time-*t*, solution to

(A12)
$$\frac{dD_{t}\left(i\right)}{D_{t}\left(i\right)}=g_{t}\left(i\right)dt+\sigma_{\mathrm{dt}}\left(i\right)dZ_{t}\left(i\right),$$

where $dZ_{t}(i)$ is a string, and $g_{t}(i)$ and $\sigma_{\mathrm{dt}}(i)$ are asset-*i* dividend growth and volatility. We denote the string correlation function with ρ(⋅,⋅), as usual.

We assume that there is a single agent with instantaneous utility and constant relative risk aversion equal to *γ*, and subjective discount rate equal to *δ*. The model may well be extended throughout more general specifications of preferences, including habit formation.

*Aggregate consumption and pricing kernel*. Denote the aggregate dividends with $D_{t}\equiv \int_{0}^{1}D_{t}(i)\;di$. They satisfy

(A13)
$$\frac{dD_{t}}{D_{t}}=\left(\int_{0}^{1}g_{t}\left(i\right)s_{t}\left(i\right)di\right)dt+\int_{0}^{1}\sigma_{\mathrm{dt}}\left(i\right)s_{t}\left(i\right)dZ_{t}\left(i\right)di,$$

where $s_{t}(i)\equiv \frac{D_{t}(i)}{D_{t}}$ denotes the ‘dividend share’ of asset-*i*. In equilibrium, aggregate dividends equal aggregate consumption, $C_{t}$ say, i.e. $D_{t}=C_{t}$. By Itô's lemma, $s_{t}(i)$ satisfies

(A14)
$$\frac{ds_{t}\left(i\right)}{s_{t}\left(i\right)}=\mu_{t}^{s}\left(i\right)dt+\sigma_{\mathrm{dt}}\left(i\right)dZ_{t}\left(i\right)-\int_{0}^{1}\sigma_{\mathrm{dt}}\left(j\right)s_{t}\left(j\right)dZ_{t}\left(j\right)dj,$$

where, denoting $\sigma_{\mathrm{dt}}^{2}\;dt\equiv \mathrm{va}r_{t}(\frac{dD_{t}}{D_{t}})$ and $\mathrm{co}v_{d_{i}d,t}\;dt\equiv \mathrm{co}v_{t}(\frac{dD_{t}(i)}{D_{t}(i)},\frac{dD_{t}}{D_{t}})$,

$$\begin{array}{rl} & \mu_{t}^{s}\left(i\right)=g_{t}\left(i\right)-\int_{0}^{1}g_{t}\left(i\right)s_{t}\left(i\right)di+\sigma_{\mathrm{dt}}^{2}-\mathrm{co}v_{d_{i},d,t},\\ & \sigma_{\mathrm{dt}}^{2}=\iint_{i,j\in {\left[0,1\right]}^{2}}\sigma_{\mathrm{dt}}\left(i\right)s_{t}\left(i\right)\rho \left(i,j\right)\sigma_{\mathrm{dt}}\left(j\right)s_{t}\left(j\right)di\;dj,\\ & \mathrm{co}v_{d_{i}d,t}=\sigma_{\mathrm{dt}}\left(i\right)\int_{0}^{1}\sigma_{\mathrm{dt}}\left(j\right)s_{t}\left(j\right)\rho \left(i,j\right)dj. \end{array}$$

It is easy to see that $\int_{0}^{1}\mu_{t}^{s}(i)s_{t}(i)\;di=0$, such that $\int_{0}^{1}\;ds_{t}(i)=0$.

In this economy, the stochastic discounting factor is $\xi_{t}=e^{-\mathrm{\delta t}}C_{t}^{-\gamma }$. It satisfies

(A15)
$$\frac{d\xi_{t}}{\xi_{t}}=-r\;dt-\int_{0}^{1}\phi \left(s_{t}\left(j\right)\right)dZ_{t}\left(j\right)dj,\;\phi \left(s_{t}\left(j\right)\right)=\gamma \sigma_{\mathrm{dt}}\left(j\right)s_{t}\left(j\right).$$

Note that $\phi (s_{t}(j))$ in equation (A15) is the compensation required to hold any asset, the returns of which are exposed to co-movements with the dividends paid by asset-*j*. Due to the nature of the model that we are dealing with (consumption-CAPM), the functional form for ϕ(⋅) in equation (A15) resembles that of the unit risk-premia arising within a multi-asset Brownian economy (see Mele (2022, equation (9.120))). Because the model in this section relies on strings, this analogy is only formal: in the model of this appendix, the unit premium for asset dividend-*j* compensates for the risk that any other asset returns are exposed to; in the Brownian model, unit premia compensate for each Brownian motion that asset returns are exposed to.

*Asset returns*. We make a few simplifying assumptions to render the model analytically tractable. We assume that the representative agent has log-utility, γ=1, and that the drift of each share process in (A14) is linear in $s_{t}(i)$. These assumptions lead to an affine model for the inverse of the price-dividend ratio, $p(s_{t}(i))$ say, similar as in Menzly *et al.* (2004) (MSV, in the sequel) (see equation (A17) below). Asset returns are, then, shown to be

(A16)
$$\begin{array}{rl} \frac{dP_{t}\left(i\right)+D_{t}\left(i\right)dt}{P_{t}\left(i\right)} & =E_{t}\left(i\right)dt+\;\underset{\equiv \sigma \left(s_{t},i\right)}{\underbrace{\sigma_{\mathrm{dt}}\left(i\right)\left(1+\frac{p^{\prime }\left(s_{t}\left(i\right)\right)}{p\left(s_{t}\left(i\right)\right)}s_{t}\left(i\right)\right)}}dZ_{t}\left(i\right)\\ & \;-\;\underset{\equiv -w\left(s_{t},i\right)}{\underbrace{\frac{p^{\prime }\left(s_{t}\left(i\right)\right)}{p\left(s_{t}\left(i\right)\right)}s_{t}\left(i\right)}}dZ_{t}\left(Z,s_{t}\right), \end{array}$$

where $dZ_{t}(Z,s_{t})\equiv \int_{0}^{1}\sigma_{\mathrm{dt}}(j)s_{t}(j)\;dZ_{t}(j)\;dj$, a compound string.

Equation (A16) is then a special case of equation (A10): the state is $y_{t}=s_{t}=(s_{t}(i){)}_{i\in (0,1)}$, the collection of all the share processes, and the compound string is $dZ_{t}(Z,s_{t})$, with $n(s_{t},j)=\sigma_{\mathrm{dt}}(j)s_{t}(j)$. That is, while each asset return depends on its own share process, each share process is driven by the realization of the whole string (see equation (A14)). Therefore, in equilibrium, asset returns are also driven by a compound string. In this economy, the volatility of asset-*i* returns originates from two forces. The first is the volatility of the asset dividend growth, $\sigma_{\mathrm{dt}}(i)$. The second stems from fluctuations in its price-dividend ratio, $p(s_{t}(i))$, which, in turn, originate from those in the dividend share, $s_{t}(i)$: the higher the semi-elasticity of $p(s_{t}(i))$, the more significant is this second source of volatility. The term $\sigma (s_{t},i)$ reflects both dividend volatility and price-dividend ratio volatility. Instead, $w(s_{t},i)$ only reflects price-dividend volatility. Note, also, that the price-dividend volatility determines how the realization of the compound string affects asset returns.

Expected returns on each asset, $E_{t}(i)$, can now be determined through correlations and volatility, based on equation (A11). These details are in Proposition A.2 below, and in its proof. First, we derive the price-dividend ratios in this economy under the assumptions formulated so far, and additional ones.

*Price-dividend ratios*. Consider the general formulation for the price-dividend ratio for each asset

$$p\left(s_{t},i\right)\equiv \frac{P_{t}\left(i\right)}{D_{t}\left(i\right)}=E_{t}\left(\int_{t}^{\infty }\frac{\xi_{u}}{\xi_{t}}\frac{D_{u}}{D_{t}}\frac{s_{u}\left(i\right)}{s_{t}\left(i\right)}du\right).$$

That is, in principle, the price-dividend ratio of any asset depends on the future paths of aggregate dividends, which, in turn, depend on the collection of all the shares process, $s_{t}$. This dimensionality problem simplifies when γ=1, in which case, the price-dividend ratio on asset-*i* only depends on the asset relative share. Under the additional assumption that, in (A14), $\mu_{t}^{s}(i)s_{t}(i)=\beta ({\bar{s}}_{i}-s_{t}(i))$, for some $({\bar{s}}_{i}{)}_{i\in (0,1)}$ and *β*, we have that $p(s_{t}(i))\equiv p(s_{t},i)$, where

(A17)
$$p\left(s_{t}\left(i\right)\right)=\frac{1}{\delta +\beta }+\frac{\beta }{\delta \left(\delta +\beta \right)}\frac{{\bar{s}}_{i}}{s_{t}\left(i\right)}.$$

The constants $({\bar{s}}_{i}{)}_{i\in (0,1)}$ satisfy $\int_{0}^{1}{\bar{s}}_{j}\;dj=1$, and *β* is constant in time and across assets, such that the shares sum up to one for all *t*. Note that the price-dividend ratio has the same functional form as in MSV. However, the model implications on the correlation of asset returns and the cross-section of expected returns are distinct, as we now explain.

Let $\mathrm{co}v_{s(i),d,t}\;dt\equiv \mathrm{cov}(\frac{ds_{t}(i)}{s_{t}(i)},\frac{dD_{t}}{D_{t}})$ and $\mathrm{co}v_{s_{i},d_{j},t}\;dt\equiv \mathrm{cov}(\frac{ds_{t}(i)}{s_{t}(i)},\frac{dD_{t}(j)}{D_{t}(j)})$. We have:

Proposition A.2 (Correlation and expected returns) We have

(A18)
$$E_{t}\left(s_{t},i\right)-r\left(s_{t}\right)=\sigma_{\mathrm{dt}}^{2}+\frac{1}{1+\frac{\beta }{\delta }\frac{{\bar{s}}_{i}}{s_{t}\left(i\right)}}\mathrm{co}v_{s_{i},d,t},$$

where

(A19)
$$\begin{array}{rl} & \mathrm{co}v_{s_{i},d,t}\\ & =\int_{0}^{1}s_{t}\left(j\right)\underset{=\mathrm{co}v_{s_{i},d_{j},t}}{\underbrace{\sigma_{\mathrm{dt}}\left(j\right)\left(\sigma_{\mathrm{dt}}\left(i\right)\rho \left(i,j\right)-\int_{0}^{1}\rho \left(j,u\right)\sigma_{\mathrm{dt}}\left(u\right)s_{t}\left(u\right)du\right)}}\;dj. \end{array}$$

Proof. By equation (A11), and the expression for the unit prices of risk, $\phi (s_{t}(j))=\sigma_{\mathrm{dt}}(j)s_{t}(j)$, the cross-section of expected excess returns is

(A20)
$$\begin{array}{rl} & E_{t}\left(s_{t},i\right)-r\left(s_{t}\right)\\ & =\sigma \left(s_{t},i\right)\int_{0}^{1}\sigma_{\mathrm{dt}}\left(j\right)s_{t}\left(j\right)\rho \left(i,j\right)dj\\ & \;+w\left(s_{t},i\right)\underset{=\sigma_{\mathrm{dt}}^{2}}{\underbrace{\iint_{u,v\in {\left[0,1\right]}^{2}}\sigma_{\mathrm{dt}}\left(u\right)s_{t}\left(u\right)n\left(s_{t},v\right)\rho \left(u,v\right)du\;dv}}, \end{array}$$

where the term indicated in the brackets coincides with $\sigma_{\mathrm{dt}}^{2}$ due to the expression of $n(s_{t},v)$ predicted by this model, $n(s_{t},v)=\sigma_{\mathrm{dt}}(v)s_{t}(v)$. Replacing the expressions for $\sigma (s_{t},i)$ (see equation (A16)) into (A20), leaves

$$\begin{array}{rl} & E_{t}\left(s_{t},i\right)-r\left(s_{t}\right)\\ & \;=\left(1-w\left(s_{t},i\right)\right)\sigma_{\mathrm{dt}}\left(i\right)\int_{0}^{1}\sigma_{\mathrm{dt}}\left(j\right)s_{t}\left(j\right)\rho \left(i,j\right)dj\\ & \;+\left(1+w\left(s_{t},i\right)-1\right)\sigma_{\mathrm{dt}}^{2}\\ & \;=\sigma_{\mathrm{dt}}^{2}+\left(1-w\left(s_{t},i\right)\right)\left(\sigma_{\mathrm{dt}}\left(i\right)\int_{0}^{1}\sigma_{\mathrm{dt}}\left(j\right)s_{t}\left(j\right)\rho \left(i,j\right)dj-\sigma_{\mathrm{dt}}^{2}\right)\\ & \;=\sigma_{\mathrm{dt}}^{2}+\left(1-w\left(s_{t},i\right)\right)(\int_{0}^{1}\sigma_{\mathrm{dt}}\left(j\right)s_{t}\left(j\right)(\sigma_{\mathrm{dt}}\left(i\right)\rho \left(i,j\right)\\ & \;-\int_{0}^{1}\rho \left(j,u\right)\sigma_{\mathrm{dt}}\left(u\right)s_{t}\left(u\right)du)dj), \end{array}$$

where the last line follows by the expression for $\sigma_{\mathrm{dt}}^{2}$. equation (A18) follows by the definition of $w(s_{t},i)$ and by a direct calculation.

The first term on the R.H.S. of equation (A18), $\sigma_{\mathrm{dt}}^{2}$, is the standard single Lucas' tree prediction. The second term can take either sign. For any asset-*i* such that the values of $\mathrm{co}v_{s_{i},d_{j},t}$ across *j* make this second term positive, the expected excess returns are increasing in $s_{t}(i)$. Intuitively, asset-*i* is not a good hedge if its share is positively correlated with a sufficiently large set of the assets' dividends. In this case, the expected return on asset-*i* is increasing in $s_{t}(i)$, as this asset pays a larger portion of consumption. This conclusion is reversed (that is, the second term of the R.H.S. in (A18) is negative) when the covariance between asset-*i* share is negatively correlated with a sufficiently large set of the assets' dividends.

Note that in multi-asset economies with dividends driven by Brownian motions, expected excess returns that go beyond the standard Lucas' tree prediction are also explained by the covariance of the share process with aggregate consumption, just as in (A18) (see Mele (2022, equation (9.129), p. 502)). However, the Brownian model does not disentangle volatilities from correlations: if *m* and *d* denote the number of assets and Brownian motions, it is possible to show that $\mathrm{co}v_{s_{i},d,t}=\sum_{j=1}^{m}s_{t}(j)\mathrm{co}v_{s_{i},d_{j},t}$, but the covariance term,

$$\mathrm{co}v_{s_{i},d_{j},t}=v_{d,t}\left(j\right)\cdot \left(v_{d,t}\left(j\right)-\sum_{j=1}^{m}s_{t}\left(u\right)v_{d,t}\left(u\right)\right),$$

includes dividends' exposures to Brownian motions, the *d*-dimensional vectors $v_{d,t}(\cdot )$ (the counterparts to the components of $v(y_{t},\cdot )$ in equation (4) of the main text), which do not separate dividends' volatility from correlation, as in the string model (see (A19)). Finally, the model in this section is distinct from that in MSV for two reasons: (i) in our model, aggregate consumption is not I.I.D., but results from aggregation (see equation (A13 )); (ii) ours is a string model.

## Appendix 3. Additional empirical evidence

Table A1 provides parameter estimates and adjusted- $R^{2}$ of Model III (see Proposition 3.2, equation (17)) for S&P 500 sectors and index-based portfolios. Table A2 provides adjusted- $R^{2}$ for linear factor models fitted to the same portfolios in table A1.

**Table D1.** This table provides parameter estimates of $\phi_{v0}$, $\phi_{v1}$ and $\phi_{v2}$ in the time and cross-sectional variation premium Model III, $\phi (y_{t},j)=\phi_{v0}\varrho_{0}(j)+(\phi_{v1}+\phi_{v2}e^{-y_{t}})\varrho_{1}(j)$ (with t-stats below), and the pricing performance expressed as the average pricing error (*α* is annualized) across a given set of portfolios, and the fit of the model (adjusted- $R^{2}$, ${\bar{R}}^{2}$) from this regression. The first column provides the number of stocks in each portfolio (*J*). The last line provides the average adjusted- $R^{2}$ across all portfolios for each model.

	*J*	$\phi_{v0}$	$\phi_{v1}$	$\phi_{v2}$	*α*	${\bar{R}}^{2}$
ENERGY	34	−34.458	19.024	−1.129	0.055	0.513
		−2.890	1.937	−0.113	1.612	–
MATERIALS	32	−76.475	37.816	−5.737	0.110	0.517
		−1.763	1.350	−0.274	4.712	–
INDUSTRIALS	76	132.112	−70.574	41.356	0.073	0.181
		1.700	−1.799	2.354	2.303	–
CONS. DISCRET.	79	51.391	−28.591	20.876	0.086	0.088
		0.969	−0.931	0.926	3.674	–
CONS. STAPLES	35	−98.597	24.520	35.578	0.294	0.523
		−1.229	0.462	0.775	4.318	–
HEALTH	48	−62.965	30.130	−1.497	0.003	0.401
		−0.747	0.610	−0.046	0.070	–
FINANCIALS	71	−30.543	13.839	−1.601	0.116	0.636
		−6.684	3.863	−0.308	7.081	–
TECHNOLOGY	51	49.876	−43.341	50.427	0.108	0.189
		2.253	−2.674	3.060	2.119	–
UTILITIES	28	−15.420	11.473	−13.129	0.179	0.374
		−1.055	0.823	−0.866	10.235	–
SP 100	107	−14.767	2.637	8.127	0.102	0.049
		−0.892	0.229	0.653	5.905	–
DOW JONES 30	37	48.786	-46.909	60.455	0.071	0.151
		1.761	−2.344	2.737	2.023	–
NASDAQ 100	109	−41.579	7.477	25.557	0.052	0.256
		−1.616	0.551	2.732	1.959	–
Average ${\bar{R}}^{2}$						0.429

**Table D2.** This table provides adjusted- $R^{2}$ from linear factor model regressions across the portfolios in Table A1. The first column provides the number of stocks in each portfolio (*J*). The second through the fourth provide adjusted- $R^{2}$ for the CAPM, the 3-F model (market, value, and size), the 4-F model (market, value, size, and momentum), and the 5-F model (market, size, value, profitability, and investment factors). The last line provides the average adjusted- $R^{2}$ across all portfolios for each model.

	*J*	CAPM	3-F	4-F	5-F
ENERGY	34	0.216	0.311	0.431	0.344
MATERIALS	32	0.092	0.214	0.306	0.347
INDUSTRIALS	76	0.079	0.157	0.449	0.350
CONS. DISCRET.	79	−0.001	0.210	0.345	0.266
CONS. STAPLES	35	−0.028	0.143	0.408	0.184
HEALTH	48	0.492	0.633	0.731	0.668
FINANCIALS	71	0.026	0.247	0.299	0.267
TECHNOLOGY	51	0.134	0.305	0.506	0.386
UTILITIES	28	0.677	0.650	0.748	0.750
SP 100	107	0.092	0.313	0.524	0.297
DOW JONES 30	37	−0.020	0.110	0.371	0.342
NASDAQ 100	109	0.074	0.130	0.346	0.224
Average ${\bar{R}}^{2}$	–	0.178	0.226	0.436	0.379
